# A Versatile Strategy for the Synthesis of 4,5-Dihydroxy-2,3-Pentanedione (DPD) and Related Compounds as Potential Modulators of Bacterial Quorum Sensing

**DOI:** 10.3390/molecules23102545

**Published:** 2018-10-06

**Authors:** Silvia Stotani, Viviana Gatta, Federico Medda, Mohan Padmanaban, Anna Karawajczyk, Päivi Tammela, Fabrizio Giordanetto, Dimitrios Tzalis, Simona Collina

**Affiliations:** 1 Medicinal Chemistry, Taros Chemicals GmbH & Co. KG, Emil-Figge-Straße 76a, 44227 Dortmund, Germany; silviastotani@hotmail.it (S.S.); fmedda@centurionbiopharma.com (F.M.); mpadmanaban@taros.de (M.P.); akarawajczyk@gmail.com (A.K.); Fabrizio.Giordanetto@deshawresearch.com (F.G.); dtzalis@taros.de (D.T.); 2 Department of Drug Sciences, Medicinal Chemistry and Pharmaceutical Technology Section, University of Pavia, Viale Taramelli 12, 27100 Pavia, Italy; 3 Drug Research Program, Division of Pharmaceutical Biosciences, Faculty of Pharmacy, University of Helsinki, FI-00014 Helsinki, Finland; viviana.gatta@helsinki.fi (V.G.); paivi.tammela@helsinki.fi (P.T.)

**Keywords:** antibiotic resistance, quorum sensing, DPD, DPD-related compounds

## Abstract

Resistance to antibiotics is an increasingly serious threat to global public health and its management translates to significant health care costs. The validation of new Gram-negative antibacterial targets as sources for potential new antibiotics remains a challenge for all the scientists working in this field. The interference with bacterial Quorum Sensing (QS) mechanisms represents a potentially interesting approach to control bacterial growth and pursue the next generation of antimicrobials. In this context, our research is focused on the discovery of novel compounds structurally related to (*S*)-4,5-dihydroxy-2,3-pentanedione, commonly known as (*S*)-DPD, a small signaling molecule able to modulate bacterial QS in both Gram-negative and Gram-positive bacteria. In this study, a practical and versatile synthesis of racemic DPD is presented. Compared to previously reported syntheses, the proposed strategy is short and robust: it requires only one purification step and avoids the use of expensive or hazardous starting materials as well as the use of specific equipment. It is therefore well suited to the synthesis of derivatives for pharmaceutical research, as demonstrated by four series of novel DPD-related compounds described herein.

## 1. Introduction

Bacterial chemical communication (i.e., quorum sensing, QS) allows bacteria to coordinate their gene expression and act as a population [1,2,3,4,5]. This phenomenon is detrimental for humans as QS regulates pathogenic processes such as the virulence factor production [6,7], susceptibility to antibiotics [8] and biofilm formation [9,10,11]. In recent decades, the modulation of QS has therefore emerged as a potential therapeutic approach to fight bacterial infections [12,13,14,15,16,17].

**Figure 1:**
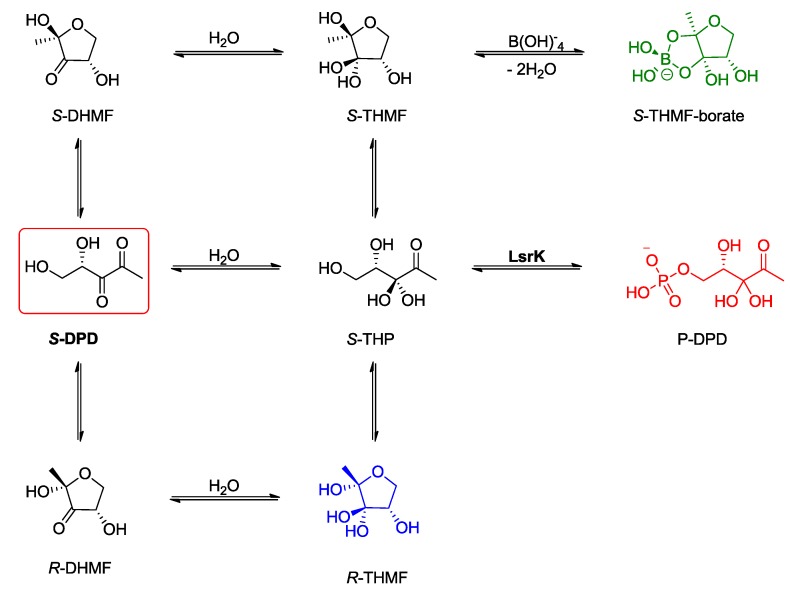
(*S*)-DPD in an aqueous medium: all species in equilibrium. (2*S*,4*S*)-2,4-dihydroxy-2-methyldihydrofuran-3-one (*S*-DHMF); (*S*)-4,5-dihydroxy-2,3-pentanedione (DPD); (2*R*,4*S*)-2,4-dihydroxy-2-methyldihydrofuran-3-one (*R*-DHMF); (2*S*,4*S*)-2-methyl-2,3,3,4-tetrahydroxytetrahydrofuran (*S* THMF); (*S*)-3,3,4,5-tetrahydroxy-2-pentanone (*S*-THP); (2*R*,4*S*)-2-methyl-2,3,3,4-tetrahydroxytetrahydrofuran (*R*-THMF); (2*S*,4*S*)-2-methyl-2,3,3,4-tetrahydroxytetrahydrofuranborate (*S*-THMF-borate); (*S*)-3,3,4,5-tetrahydroxy-2-pentanone-5-phosphate (P-DPD).

QS is mediated by production and release of and response to small molecules called autoinducers (AIs). Among these AIs, Autoinducer-2 (AI-2) is responsible for intra- and interspecies bacterial communication and, as a consequence, it has been termed the “universal autoinducer”. The development of small molecules able to modulate the AI-2-mediated signaling would possibly result in broad-spectrum antimicrobial activity. However, targeting the AI-2-based QS remains challenging mostly because of the rapid interconversion of the AI-2 precursor (*S*)-DPD (Figure 1) to several linear and cyclic forms recognized by different bacteria [18] (Figure 1). In aqueous solutions, (*S*)-DPD is in equilibrium with its two cyclic stereoisomers (*S*-DHMF and *R*-DHMF; Figure 1) [19]. Hydration of the C_3_ carbonyl group of both the cyclic and linear structures was confirmed by X-ray crystallography. In the presence of boric acid, *S*-THMF (Figure 1) forms a borate ester (*S*-THMF-borate; Figure 1) which is recognized by LuxP in *V. harveyi* (PDB ID: 1JX6) [20]. *R*-THMF instead (Figure 1) does not coordinate boron and binds to the transporter LsrB which is responsible for its internalization and acts as the active species in *S. thyphimurium* AI-2-mediated QS (PDB ID: 1TJY) [21]. The hydrated form of linear (*S*)-DPD (*S*-THP, Figure 1) is phosphorylated by LsrK, resulting in phospho-DPD (P-DPD, Figure 1) [22] recognized by the transcriptional repressor LsrR (PDB ID: 4L4Z) [23] and responsible for *E coli* and *S. typhimurium* AI-2-mediated signaling.

Modulation/inhibition of QS can control several bacterial virulence factors (e.g., biofilm formation) that facilitate human infections and reduce their negative effects, including mortality [24]. Quorum Sensing Inhibitors (QSI) therefore represent interesting tools to use in combination with “conventional” antibiotic therapies against antimicrobial resistance (AMR) [25,26].

**Figure 2:**
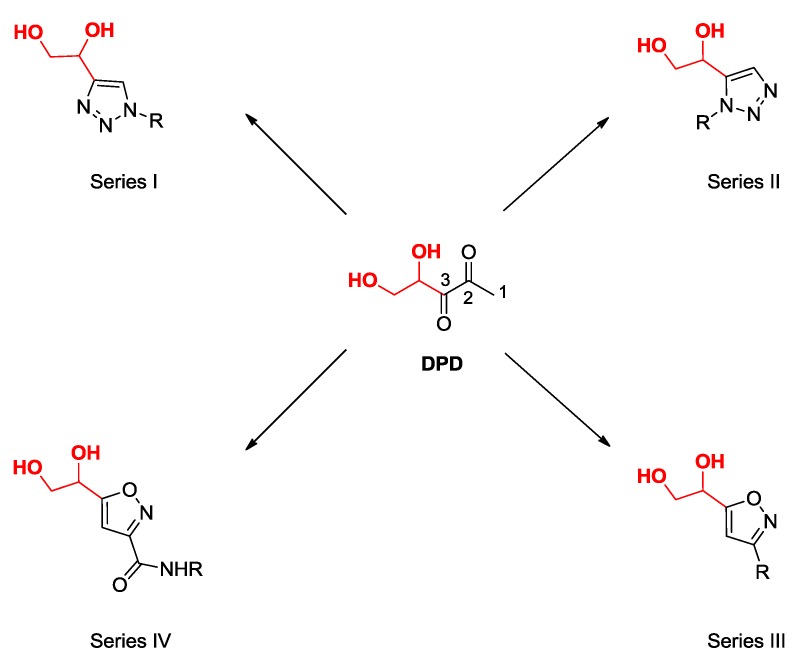
The DPD-related compounds presented in this work.

In this work, we describe the set-up of a new protocol for the synthesis of racemic DPD and its application to the synthesis of four novel small libraries of DPD-related compounds (Figure 2), designed to target LsrK kinase, a key mediator in AI-2-mediated QS in enteric bacteria. The essential role of the enzyme has been demonstrated by LsrK gene deletion in *E. coli*, generating a mutant strain unable to activate AI-2-mediated QS [27]. Therefore, we believe that the generation of DPD-related compounds for the inhibition of LsrK may be the starting point for the development of new QSI that will serve as potential tools for overcoming antimicrobial resistance.

## 2. Results and Discussion

Since 2004, much effort has been devoted to the study of synthetic pathways for the preparation of DPD and analogs in both racemic and enantiomeric forms. Literature analysis revealed that the synthesis of homochiral DPD requires the use of expensive (i.e., (*S*)-1,4-dioxaspiro[4.5]decane-2-carboxaldehyde) or unstable (i.e., (*S*)-glyceraldehyde acetonide) chiral starting materials and of further time-consuming purification steps [18,28,29,30,31,32,33]. Conversely, the synthetic procedures published so far to obtain racemic DPD proceed smoothly but suffer from hazardous chemical steps (i.e., reductive ozonolysis or the use of diazomethane) [34,35].

Starting from these considerations and keeping in mind that in the initial phase of the drug discovery process racemic compounds are usually evaluated and only once the most active ones have been identified both enantiomers must be prepared for biological testing [36], herein we studied a novel versatile strategy for the synthesis of racemic DPD suitable for readily supporting practical chemical diversification. The proposed synthetic strategy leading to DPD could be useful for the preparation of C_1_ DPD-analogs and for the synthesis of DPD structurally related compounds, where the two carbonyl groups of DPD at C_2_ and C_3_ are embedded in heteroaromatic rings (Figure 2). To the best of our knowledge, no modification at C_2_ have been reported and position C_3_ has been barely explored and no heteroaromatic substituents (except for a furan at C_1_) were previously described.

### 2.1. Synthesis of DPD and Ph-DPD

**Scheme 1:**
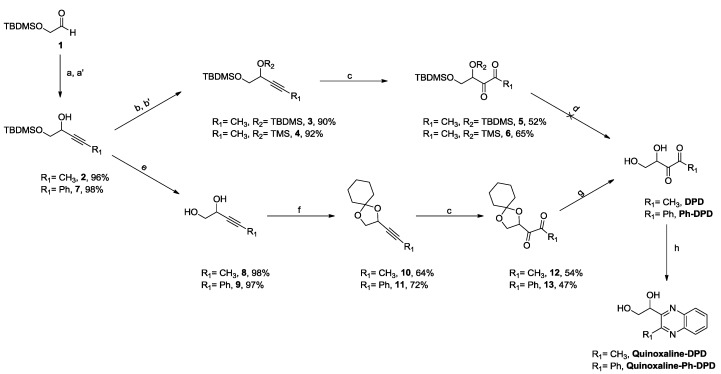
The synthesis of racemic DPD, Ph-DPD, quinoxaline-DPD and quinoxaline-Ph-DPD. Reagents and conditions: (a) 1-propynylmagnesium bromide (0.5 M in THF, 1.3 eq), THF, 0 °C to rt, 3 h; (a′) phenylethynylmagnesium bromide (1.0 M in THF, 1.3 eq), THF, 0 °C to rt, 3 h; (b) TBDMSCl (1.2 eq), NaH (2.0 eq), THF, rt, 3 h; (b′) TMSCl (1.2 eq), NaH (2.0 eq), THF, rt, 3 h; (**c**) NaIO_4_ (4.4 eq), Ru_2_O·H_2_O (2.5% mol), CHCl_3_/ACN/H_2_O (1:1:1), rt, 1 h; (d) see Table S1; (e) Dowex50WX8 100–200 mesh, MeOH, rt, overnight; (f) cyclohexanone dimethyl ketal (3.0 eq), *p*-TSA (cat.), rt, overnight; (g) Dowex50WX8 100–200 mesh, D_2_O (10 mM), rt, overnight; (h) *o*-phenylendiamine (2.0 eq), rt, overnight.

The synthetic strategies originally evaluated are outlined in Scheme 1.

Briefly, the addition of 1-propynylmagnesium bromide to (*t*-butyldimethylsilyloxy)acetaldehyde [37] (**1**, Scheme 1), followed by the protection of the resulting secondary alcohol with TBDMSCl or TMSCl afforded compounds **3** or **4**, respectively (Scheme 1). The subsequent oxidation of the internal alkyne to yield diketone **5** or **6** was performed under optimized RuO_2_*H_2_O/NaIO_4_-catalyzed conditions (Table 1, entry 5) using CHCl_3_/ACN/H_2_O (1:1:1) as the solvent.

The final acidic removal of the two TBDMS groups of compound **5** was performed under different conditions, but resulted in being unsuccessful (Table S1). Particularly, decomposition was observed when H_2_SO_4_ (or D_2_SO_4_) and TBAF were employed (Table S1). The partial removal of the two protecting groups (up to a maximum of 30% in total) was achieved with the use of acetic acid or Dowex50WX8 (Table S1). When the bulky protecting TBDMS group was replaced with TMS, (Scheme 1), similar results were obtained and a maximum of 40% cleavage was achieved using Dowex50WX8 in ACN-*d*_3_.

A different approach was then investigated: compound **2** and the analogous **7** were deprotected in acidic conditions (Dowex50WX8), affording diols **8** and **9**, respectively. These intermediates were then protected with a cyclohexyliden group and oxidized under the previously described conditions (Table 1). The oxidation of **10** and **11** was followed by the Dowex50WX8-mediated removal of the protecting group. ^1^H-NMR analysis of the crude products was consistent with the literature-reported data and revealed the presence of a mixture of structures in equilibrium with each other (see SI for additional details). To further confirm the success of our procedure, the mixtures were treated with *o*-phenilendiamine to form, respectively, quinoxaline-DPD and quinoxaline-Ph-DPD (Scheme 1), which were isolated and fully characterized.

To sum up, the approach described above allows for the rapid production of racemic DPD in five steps and it does not require the use of dangerous or expensive reagents nor of particular equipment (i.e., ozonolysator); furthermore, only one purification step via column chromatography is necessary. Not less important, this procedure is suitable for the synthesis of C_1_-DPD analogs (as long as the corresponding Grignard reagent can be purchased or produced) as the synthesis of Ph-DPD demonstrated. Additionally, the ethyne function introduced in the first step is a practical synthetic handle for further chemical derivatization, as demonstrated by the four small series of derivatives described below.

### 2.2. Synthesis of DPD-Related Compounds

As anticipated, we designed novel DPD-related compounds in which the carbonyl groups at C_2_ and C_3_ are embedded in heteroaromatic moiety to obtain compounds stable in solution, thus avoiding the open/closed equilibrium typical of the majority of the DPD-analogs reported so far (Figure 1). As heteroaromatic rings, we selected 1,2,3-triazole and isoxazole, two scaffolds common in medicinal chemistry present in several natural and synthetic drugs including antimicrobial, anticancer, anti-inflammatory and antireumatic drugs [38,39,40,41,42,43].

**Scheme 2:**
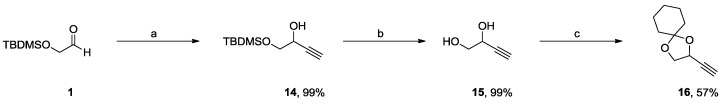
The synthesis of intermediates **15** and **16**. Reagents and conditions: (a) ethynylmagnesium bromide (0.5 M in THF, 1.3 eq), THF, 0 °C to rt, 3h; (b) Dowex50WX8 100–200 mesh, MeOH, rt, overnight; (c) cyclohexanone dimethyl ketal (3.0 eq), *p*-TSA (cat.), rt, overnight.

The newly designed compounds can be obtained starting from the two common intermediates **15** and **16** (Scheme 2) strictly related to **2** and **7** (Scheme 1). In details, as in the case of DPD, the first of the two building blocks necessary to start the synthesis of all the analogs presented in this work was produced by the Grignard addition of ethynylmagnesium bromide to aldehyde **1**, followed by acidic removal of the TBDMS protecting group. Further protection of the resulting diol **15** as acetal, using cyclohexanone dimethyl ketal, afforded the second building block compound **16** (Scheme 2).

#### 2.2.1. 1,4- and 1,5-Disubstituted 1,2,3-Triazoles DPD-Derivatives (Series I and II)

1,2,3-triazoles (both 1,4- and 1,5-disubstituted) can be synthesized applying azide-alkyne Huisgen cycloaddition conditions where an azide is reacted with an alkyne in a 1,3-dipolar cycloaddition reaction. At first, we tested three different Copper-Catalyzed Azide-Alkyne Cycloaddition (CuAAC) conditions to select the best procedure for the synthesis of the planned compounds. For this purpose, (2-azidoethyl)benzene (**17a**) was chosen as the reference azide (Table 2). First, we used CuI (10% mol) and DIPEA (15% mol) in nonaqueous, nonprotic THF to afford the desired product with 58% isolated yield (Table 2, entry 1) [44]. As the addition of AcOH was found to accelerate the protonation of the Cu-C bond [45,46,47] (thus facilitating the formation of the product), a catalytic amount of AcOH was added to the mixture (containing 2% mol CuI and 4% mol DIPEA). This acid-base system jointly promoted CuAAC and resulted in a 14% increase of the isolated yield (Table 2, entry 2) when compared to the previous conditions (Table 2, entry 1) [48]. It is known that the use of ligands is beneficial for the reaction as it prevents Cu(I) oxidation and avoids the use of a base. Therefore, it is not surprising that the in situ generation of Cu(I) by the reduction of CuSO_4_*5H_2_O from sodium ascorbate together with the formation of L-ascorbic acid (that acts both as a ligand and as acidic source) raised the yield up to 89% (Table 2, entry 3) [49]. The 1,4-disubstitution was confirmed by the HMBC of compound **18a** (see Supporting Information).

The corresponding 1,5-disubstituted 1,2,3-triazole **19a** was also synthesized by varying the experimental conditions: the regioselective synthesis was achieved with the use of Ruthenium-catalyzed Azide-Alkyne Cycloaddition (RuAAC) conditions. Azide **17a** was reacted with terminal alkyne **15** in the presence of 2% mol pentamethylcyclopentadienylbis (triphenylphosphine)ruthenium(II) chloride (Cp*RuCl(PPh_3_)_2_) regioselectively yielding, after stirring overnight the mixture in refluxing 1,4-dioxane, the corresponding 1,5-disubstituted 1,2,3-triazole **19a** (Table 2, entry 4). ^1^H, ^13^C, TLC, UHPLC, and HMBC unambiguously confirmed the different nature of the two compounds (see Supporting Information) [50].

Once optimal conditions for the regioselective synthesis of 1,4-disubstituted 1,2,3-triazoles were established, we synthesized five azides of different chemical nature including aromatic, heteroaromatic and aliphatic elements (**17b**–**f**). This was achieved by stirring overnight at room temperature the corresponding bromo compounds with an excess (1.5 eq) of sodium azide. The five azides were reacted with alkyne **15** applying the previously found conditions and products **18b**–**f** were isolated in good to excellent yields (60–88%, Table 2, entry 5–9).

**Scheme 3:**
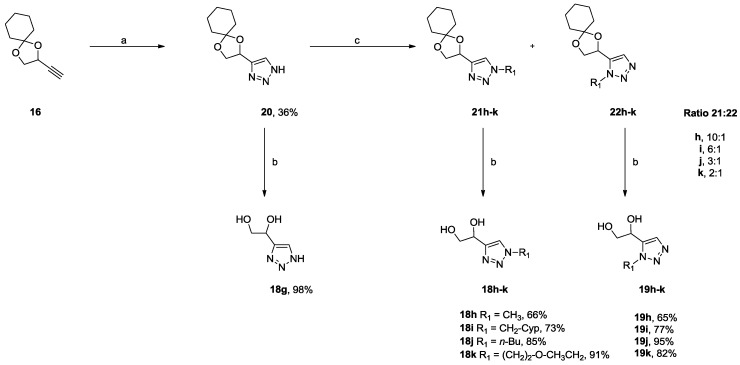
The synthesis of 1,4-disubstituted 1,2,3-triazoles **18g**–**k** and 1,5-disubstituted 1,2,3-triazoles **19h**–**k**. Reagents and conditions: (a) TMSN_3_ (10.0 eq), CuSO_4_·5H_2_O (5% mol), Na ascorbate (0.5 eq), *t*-BuOH/H_2_O (1:1), rt, overnight; (b) 12M HCl (cat.), 1,4-dioxane, 0 °C to rt, 1–3 h; (c) R_1_Br (1.5 eq), K_2_CO_3_ (2.0 eq), THF, 40 °C, overnight; preparative HPLC.

As the synthesis of triazoles substituted with short alkyl chains (e.g., methyl, butyl) was unattainable by this route because of safety issues related to the explosive and unstable nature of the required azides, we installed the desired substituents on the triazole scaffold via alkylation. We elected to use a single, small and dangerous azide (i.e., TMSN_3_) over the use of four different ones. The acetal protected terminal alkyne **16** was carefully reacted with an excess (10.0 eq) of TMSN_3_ under previously established CuAAC conditions. The resulting unsubstituted triazole (**20**, Scheme 3) was both deprotected under acidic conditions (**18g**, Scheme 3) and, to install the desired substituents, alkylated with four different (i.e., methyl, cyclopropylmethyl, butyl, ethoxyethyl) bromides (Scheme 3).

As expected, no regioselectivity was observed and both the 1,4- and the 1,5-disubstituted 1,2,3-triazoles formed. Experimenting with base (i.e, 1.1 eq, 1.3 eq and 1.5 eq of K_2_CO_3_) and/or the alkylbromides (i.e., 0.8 eq and 0.9 eq of R_1_Br) stoichiometry did not consistently changed the ratio of the two regioisomers (data not shown). For each substituent, the two corresponding regioisomers were isolated by preparative HPLC. The resulting eight products (**21h**–**k** and **22h**–**k**, Scheme 3) were lastly deprotected with a catalytic amount of concentrated hydrochloric acid. The ratio of the two regioisomers was determined by crude NMR. For all of the four regioisomeric pairs, the 1,4-dibustituted 1,2,3-triazoles formed in excess when compared to the respective 1,5-regioisomers and, as predictable, the ratio decreased as the sterical hindrance of the R_1_ substituent increased (Scheme 3). Concentrated HCl was preferred over Dowex 50WX8 for the removal of the acetal protecting group due to the shorter reaction time (1–3 h vs. overnight) and shorter workup (no filtration to remove the acidic resin required).

#### 2.2.2. 3,5-Disubstituted Isoxazoles DPD-Derivatives (Series III and IV)

**Scheme 4:**
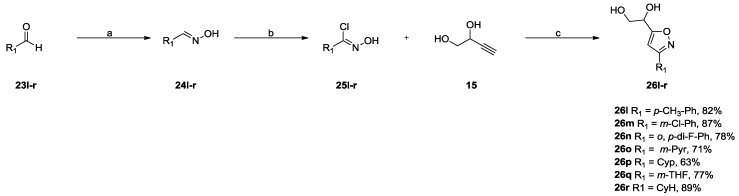
The synthesis of 3,5-disubstituted isoxazoles **26l**–**r**. Reagents and conditions: (a) NH_2_OH·HCl (3.0 eq), Et_3_N (1.5 eq), H_2_O/EtOH (1:1), rt, 1–3 h; (b) NCS (1.0 eq), DMF, rt, 1–2 h; (c) 15 (1.0 eq), CuSO_4_·5H_2_O (5% mol), Na ascorbate (0.5 eq), KHCO_3_, *t*-BuOH/H_2_O (1:1), rt, overnight.

Compound **15** (Scheme 2) is also the key intermediate for the synthesis of 3,5-disubstituded DPD related compounds **26l**–**r** (Scheme 4). Briefly, aldehydes **23l**–**r** were converted into their corresponding oximes **24l**–**r** using NH_2_OH·HCl. The resulting crude compounds were directly chlorinated by a reaction with *N*-chlorosuccinimide (NCS). According to Himo et al. [49], the addition of CuSO_4_·5H_2_O, Na ascorbate, and KHCO_3_ in *t*-BuOH/H_2_O (1:1) to the isolated chloro-oximes allowed them to form the nitrile oxide which reacted by 1,3-dipolar cycloaddition with **15**. After preparative HPLC purification, the targeted isoxazoles **26l**–**r** were, therefore, obtained in good to excellent yields (i.e., 63–89%, Scheme 4).

**Scheme 5:**
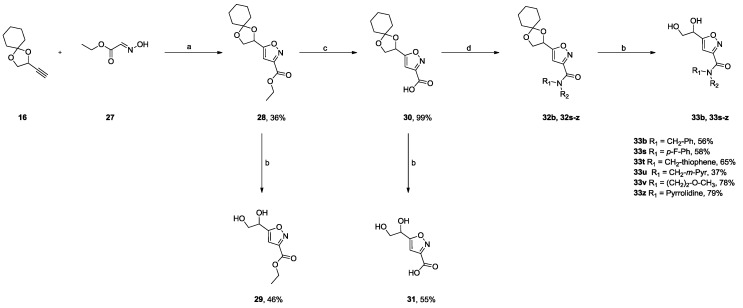
The synthesis of 3,5-disubstituted isoxazoles (with an amide moiety at position 3) **33b**, **33s–z**. Reagents and conditions: (a) 27 (2.0 eq), NaOCl (40.0 eq), THF, rt, 12 h; (b) 12M HCl (cat.), 1,4-dioxane, 0 °C to rt, 1–3 h; (c) NaOH 1M (3.0 eq), THF, rt, overnight; (d) amine (2.0 eq), HOBt (2.0 eq), EDC*HCl (2.0 eq), Et_3_N, DCM, rt, overnight.

The same procedure was attempted to obtain 3,5-disubstituted isoxazoles of DPD-analogs bearing an amide moiety at position 3, but starting from the protected precursor **16** instead of **15** due to the cross-reactivity between the 1,3-diol and the reagents necessary in the following steps (e.g., NaOH, DIPEA, Scheme 5). Formation of the nitrile oxide for the cycloaddition was attempted using the dehydration of ethyl nitroacetate with several bases (i.e., DABCO, DMAP, DBU, NMI, Scheme S1, conditions a) and also with a combination of PhNCO/Et_3_N (Scheme S1, conditions b), commonly used to activate nitro groups. All of the aforementioned methods resulted in a mixture of unreacted starting materials [51,52].

The 1,3-dipole species was then changed to the chloro-oxime of ethyl glyoxalate (50% solution in toluene) but the employment of the same conditions as above (CuSO_4_·5H_2_O (5% mol), Na ascorbate (0.5 eq), KHCO_3_, *t*-BuOH/H_2_O (1:1), Scheme 4) did not yield the desired product while the simple use of an equimolar amount of Et_3_N gave only traces of **28** (Scheme 5) [49,53].

We then change our strategy and employed the oxime of ethyl glyoxalate **27** together with an excess (40.0 eq) of sodium hypochlorite, both as a chlorinating agent and as a base to form the corresponding nitrile oxide, following the procedure already described by Quan et al. [54]. Compound **28** was successfully obtained, even if with a low yield (16%). Different reaction times, as well as ratios of dipolarophile **16** and 1,3-dipole **28**, were then tested (Table S2) in order to improve the initially poor yield (i.e., 16%, Table S2). Increasing the concentration of 1,3-dipole **27** enhanced the formation of intermediate **28** up to a maximum of 36% isolated yield (Table S2) with the complete consumption of the dipolarophile **16**, followed by removal of the excess of **27** by column chromatography.

Once a solution for the key 1,3-dipolar cycloaddition step was found, the rest of the synthetic pathway proceeded smoothly (Scheme 5). Saponification of the ethylic ester was followed by the amidification of the resulting carboxylic acid moiety using HOBt as the coupling agent and employing both primary and secondary amines (aromatic, heteroaromatic, aliphatic). The final acidic removal of the acetal protecting group afforded six 3,5-disubstituted isoxazoles (with an amide moiety at position 3) **33b**, **33s**–**z** in moderate to excellent yields (i.e., 37–79%, Scheme 5). Two more products were isolated after the acidic deprotection of intermediates **28** and **30** (i.e., **29** and **31**, respectively, Scheme 5).

### 2.3. Biological Evaluation of Synthetized Compounds

The activity of the synthetized compounds was evaluated with a bioluminescence-based assay against the target enzyme. Our results clearly highlight that racemic DPD prepared using our procedure is efficiently phosphorylated by LsrK (see Supporting Information, Figure S1). In fact, the level of ATP is significantly reduced by the addition of racemic DPD, resulting in a light emission lower than the sample including only LsrK and ATP.

These results confirmed the validity of the approach adopted. Indeed, in this initial phase of the drug discovery process, we prepared racemic DPD and studied a versatile synthesis suitable for readily supporting practical chemical diversification racemic compounds. Only once the most active ones have been identified will both enantiomers be prepared for biological testing. Accordingly, the activity of racemic DPD is essential for demonstrating that our approach has a valid basis. Regarding the DPD-derivatives, unfortunately, they did not show any activity (the data are reported in Supporting Information, Table S3).

## 3. Experimental

### 3.1. Chemistry

Chemicals and solvents were obtained from commercial suppliers and were used without further purification. All dry reactions were performed under a nitrogen atmosphere using commercial dry solvents. Flash column chromatography was performed on a silica column using 230-400 mesh silica gel or the Grace Reveleris X2 flash chromatography system using silica gel packed Macherey Nagel Chromabond Flash BT cartridges (60 Å, 45 μm) and Grace Reveleris flash Cartridges (60 Å, 40 μm). Thin layer chromatography was performed on Macherey Nagel precoated TLC aluminum sheets with silica gel 60 UV254 (5–17 μm). TLC visualization was accomplished by irradiation with a UV lamp (254 nm) and/or staining with KMnO_4_ solutions. ^1^H-NMR spectra were recorded at room temperature on a Bruker Avance spectrometer operating at 300 MHz (Hamburg, Germany). Chemical shifts are given in ppm (δ) from tetramethylsilane as an internal standard or residual solvent peak. Significant ^1^H-NMR data are tabulated in the following order: multiplicity (s, singlet; d, doublet; t, triplet; q, quartet; m, multiplet; dd, doublet of doublets; dt, doublet of triplets; td, triplet of doublets; br, broad), coupling constant(s) in hertz, number of protons. Proton decoupled ^13^C-NMR data were acquired at 100 MHz. ^13^C chemical shifts are reported in parts per million (δ, ppm). All NMR data were collected at room temperature (25 °C). Analytical, preparative HPLC and Electron Spray Ionization (ESI) mass spectra were performed on an Agilent UHPLC (1290 Infinity, Santa Clara, CA, USA) and an Agilent Prep-HPLC (1260 Infinity), both equipped with a Diode Array Detector and a Quadrupole MS using mixture gradients of formic acid/water/acetonitrile as solvents. High-resolution electrospray ionization mass spectra (ESI-FTMS) were recorded on a Thermo LTQ Orbitrap (Thermo Electron, Dreieich, Germany) coupled to an ‘Accela’ HPLC system supplied with a ‘Hypersil GOLD’ column (Termo Electron).

### 3.2. Synthesis of DPD and Ph-DPD

**Synthesis of 2 and 7:** to a stirred solution of (*t*-butyldimethylsilyloxy)acetaldehyde (1.0 eq) in dry THF, 1-propynylmagnesium bromide was added (over 15 min; 0.5 M in THF, 1.3 eq) (or phenylethynylmagnesium bromide (1.0 M in THF, 1.3 eq) at 0 °C). After the addition, the reaction was allowed to reach room temperature and stirred for 3 h. The solvent was removed under reduced pressure, the residue was poured into a cold saturated solution of NH_4_Cl and extracted three times with Et_2_O. The organic layer was washed twice with water and once with brine, dried over MgSO_4_, filtered and concentrated in vacuo to yield **2** as a yellowish oil (96%) or **7** as a yellow oil (98%).

*1-[(t-Butyldimethylsilyl)oxy]pent-3-yn-2-ol* (**2**): yellowish oil, 96%, *R_f_* = 0.20 (CyH/EtOAc 9:1). ^1^H-NMR (300 MHz, CDCl_3_) δ 4.36–4.34 (m, 1H), 3.73 (dd, *J* = 3.6 Hz, *J* = 10.0 Hz, 1H), 3.59 (dd, *J* = 7.7 Hz, *J* = 10.0 Hz,1H), 2.57 (s br, 1H), 1.83 (d, *J* = 1.9 Hz, 3H), 0.91 (s, 9H), 0.08 (d, *J* = 1.3 Hz, 6H) ppm; ^13^C-NMR (100 MHz, CDCl_3_) δ 81.8, 79.6, 67.3, 66.3, 25.8, 18.3, 3.5, −5.4 ppm [55].

*1-[(t-Butyldimethylsilyl)oxy]-4-phenylbut-3-yn-2-ol* (**7**): yellow oil, 98%, *R_f_* = 0.72 (CyH/EtOAc 9:1). ^1^H-NMR (300 MHz, CDCl_3_) δ 7.45–7.42 (m, 2H), 7.32–7.29 (m, 3H), 4.65–4.60 (m, 1H), 3.87 (dd, *J* = 3.8 Hz, *J* = 10.0 Hz, 1H), 3.75 (dd, *J* = 6.9 Hz, *J* = 10.0 Hz, 1H), 2.71 (d, *J* = 4.9 Hz, 1H), 0.93 (s, 9H), 0.13 (d, *J* = 3.1 Hz, 6H) ppm; ^13^C-NMR (100 MHz, CDCl_3_) δ 131.8, 128.4, 128.2, 122.5, 87.0, 85.3, 67.0, 63.6, 25.9, 18.4, 5.3 ppm [56].

**Synthesis of 8 and 9:** to a stirred solution of **2** (or **7**) (1.0 eq) in MeOH, Dowex50WX8 100–200 mesh (100 mg/1 mL) was added. The reaction was stirred at room temperature overnight. The mixture was filtered through paper and the solvent was evaporated under reduced pressure to yield **8** as an orange oil (98%) or **9** as an orange oil (97%).

*Pent-3-yne-1,2-diol* (**8**): orange oil, 98%, *R_f_* = 0.38 (CHCl_3_/MeOH 9:1). ^1^H-NMR (300 MHz, CDCl_3_) δ 4.44–4.39 (m, 1H), 3.70 (dd, *J* = 3.8 Hz, *J* = 11.3 Hz, 1H), 3.62 (dd, *J* = 6.6 Hz, *J* = 11.3 Hz, 1H), 2.41 (s br, 2H), 1.85 (d, *J* = 2.1 Hz, 3H) ppm; ^13^C-NMR (100 MHz, CDCl_3_) δ 82.8, 79.7, 66.8, 63.4, 3.5 ppm [32].

*4-Phenylbut-3-yne-1,2-diol* (**9**): orange oil, 97%, *R_f_* = 0.44 (CHCl_3_/MeOH 9:1). ^1^H-NMR (300 MHz, CDCl_3_) δ 7.46–7.42 (m, 2H), 7.36–7.29 (m, 3H), 4.69 (dd, *J* = 3.9 Hz, *J* = 6.5 Hz, 1H), 3.87–3.74 (m, 2H), 2.23 (s br, 2H) ppm; ^13^C-NMR (100 MHz, CDCl_3_) δ 131.8, 128.7, 128.3, 122.0, 86.5, 86.3, 66.6, 63.7 ppm [56].

**Synthesis of 10 and 11:** to **8** (or **9**) (1.0 eq) cyclohexanone dimethyl ketal (3.0 eq) and a catalytic amount of *p*-TSA was added. The reaction was stirred at room temperature overnight. The solvent was removed under reduced pressure and the crude was re-dissolved in Et_2_O and washed three times with NaHCO_3_. The organic layer was dried over MgSO_4_, filtered and concentrated in vacuo to yield **10** as a yellow oil (64%) or **11** as a yellow oil (72%).

*2-(Prop-1-yn-1-yl)-1,4-dioxaspiro[4.5]decane* (**10**): yellow oil, 64%, *R_f_* = 0.50 (CyH/EtOAc 9:1). ^1^H-NMR (300 MHz, CDCl_3_) δ 4.70–4.64 (m, 1H), 4.11 (dd, *J* = 6.2 Hz, *J* = 7.9 Hz, 1H), 3.81 (t, *J* = 7.5 Hz, 1H), 1.85 (d, *J* = 2.1 Hz, 3H), 1.74–1.70 (m, 2H), 1.65–1.57 (m, 6H), 1.43–1.38 (m, 2H) ppm; ^13^C-NMR (100 MHz, CDCl_3_) δ 110.5, 85.8, 82.3, 69.7, 65.5, 35.8, 25.1, 23.9, 3.7 ppm [32].

*2-(2-Phenylethynyl)-1,4-dioxaspiro[4.5]decane* (**11**): yellow oil, 72%, *R_f_* = 0.60 (CyH/EtOAc 9:1). ^1^H-NMR (300 MHz, CDCl_3_) δ 7.46–7.42 (m, 2H), 7.33–7.28 (m, 3H), 4.95 (t, *J* = 6.4 Hz, 1H), 4.23 (dd, *J* = 6.3 Hz, *J* = 7.9 Hz, 1H), 4.01 (dd, *J* = 6.5 Hz, *J* = 7.9 Hz, 1H), 1.81–1.77 (m, 2H), 1.68–1.56 (m, 6H), 1.44–1.41 (m, 2H), ppm; ^13^C-NMR (100 MHz, CDCl_3_) δ 131.8, 128.5, 128.2, 122.4, 111.0, 86.6, 85.6, 69.7, 65.7, 35.5, 25.1, 23.9 ppm.

**Synthesis of 12 and 13:** to a stirred solution of **10** (or **11**) (1.0 eq) in a 1:1:1 mixture of CHCl_3_/ACN/H_2_O, NaIO_4_ (4.4 eq) and RuO_2_·H_2_O (2.5% mol) were added. The mixture was vigorously stirred at room temperature overnight. The solvent was evaporated under reduced pressure and the crude was re-dissolved in CHCl_3_ and filtered through a silica pad. The eluate was washed three times with water, dried over MgSO_4_, filtered and concentrated in vacuo to yield **12** as a yellow oil (54%) or **13** as yellow oil (47%).

*1-{1,4-Dioxaspiro[4.5]decan-2-yl}propane-1,2-dione* (**12**): yellow oil, 54%, *R_f_* = 0.42 (CyH/EtOAc 3:1). ^1^H-NMR (300 MHz, CDCl_3_) δ 5.14 (dd, *J* = 5.3 Hz, *J* = 7.9 Hz, 1H), 4.35 (dd, *J* = 8.0 Hz, *J* = 8.9 Hz, 1H), 3.99 (dd, *J* = 5.3 Hz, *J* = 8.9 Hz, 1H), 2.39 (s, 3H), 1.66–1.57 (m, 8H), 1.45–1.42 (m, 2H) ppm; ^13^C-NMR (100 MHz, CDCl_3_) δ 197.5, 190.0, 109.2, 75.9, 66.9, 36.4, 35.6, 25.9, 24.8, 24.0 ppm [28].

*1-{1,4-Dioxaspiro[4.5]decan-2-yl}-2-phenylethane-1,2-dione* (**13**): yellow oil, 47%, *R_f_* = 0.46 (CyH/EtOAc 9:1). ^1^H-NMR (300 MHz, CDCl_3_), δ 7.98 (d, *J* = 7.1 Hz, 2H), 7.66 (d, *J* = 7.4 Hz, 1H), 7.51 (t, *J* = 7.7 Hz, 2H), 5.12 (t, *J* = 6.2 Hz, 1H), 4.34 (d, *J* = 6.4 Hz, 2H), 1.65–1.49 (m, 8H), 1.39–1.32 (m, 2H) ppm; ^13^C-NMR (100 MHz, CDCl_3_) δ 200.5, 193.0, 134.9, 132.2, 129.9, 128.9, 112.2, 77.9, 65.9, 35.4, 34.6, 24.9, 23.8, 23.0 ppm.

**Synthesis of DPD and Ph-DPD:** to a stirred solution of **12** (or **13**) (10 mM) in D_2_O, Dowex 50WX8 resin was added (100 mg/1 mL). The mixture was stirred at room temperature overnight. The mixture was filtered to remove the resin and extracted with CDCl_3_ to remove the released cyclohexanone.

*4,5-Dihydroxy-2,3-pentanedione* (**DPD**): ^1^H-NMR (300 MHz, D_2_O) δ 4.41–4.37 (m, 1H), 4.21–4.14 (m, 2H), 4.07 (dd, *J* = 3.2 Hz, *J* = 6.0 Hz, 1H), 3.99 (dd, *J* = 3.8 Hz, *J* = 7.4 Hz, 1H), 3.86–3.78 (m, 2H), 3.69–3.65 (m, 1H), 3.59 (dd, *J* = 5.6 Hz, *J* = 9.4 Hz, 1H), 2.39 (s, 3H), 1.46 (s, 3H), 1.43 (s, 3H) ppm [34]. The NMR shows that some cyclohexanone is left as two multiplets at 1.88–1.86 and 1.75–1.74 ppm.

*3,4-Dihydroxy-1-phenylbutane-1,2-dione* (**Ph-DPD**): ^1^H-NMR (300 MHz, D_2_O) δ 8.25–8.15 (m, 2H), 8.07–7.92 (m, 2H), 7.73–7.68 (m, 1H), 7.62–7.59 (m, 5H), 7.48–7.46 (m, 5H), 4.49–4.42 (m, 1H), 4.40–4.36 (m, 1H), 4.13 (dd, *J* = 2.7 Hz, *J* = 5.6 Hz, 1H), 4.09 (d, *J* = 2.8 Hz, 1H), 4.06 (d, *J* = 2.6 Hz, 1H), 3.88 (d, *J* = 4.0 Hz, 1H), 3.85–3.79 (m, 1H), 3.73–3.66 (m, 1H) ppm [30].

**Synthesis of quinoxaline-DPD and quinoxaline-Ph-DPD:** to a stirred solution of **DPD** (or **Ph-DPD**) in D_2_O, *o*-phenylendiamine (2.0 eq) was added. The reaction was stirred at room temperature overnight. The solvent was evaporated under reduced pressure, the crude was re-dissolved in ACN (1 mL), filtered and purified by preparative HPLC.

*1-(3-Methylquinoxalin-2-yl)ethane-1,2-diol* (**Quinoxaline-DPD**): orange solid, *R_f_* = 0.52 (CHCl_3_/MeOH 9:1). ^1^H-NMR (700 MHz, MeOD) δ 8.09–8.07 (m, 1H), 7.98–7.97 (m, 1H), 7.76 (pd, *J* = 7.0 Hz, *J* = 1.6 Hz, 2H), 5.15–5.13 (m, 1H), 4.02 (dd, *J* = 11.4 Hz, *J* = 5.4 Hz, 1H), 3.96 (dd, *J* = 11.4 Hz, *J* = 6.3 Hz, 1H), 2.84 (s, 3H) ppm; ^13^C-NMR (176 MHz, MeOD) δ 156.6, 154.7, 142.3, 141. 8, 131.2, 130.5, 129.9, 128.8, 72.9, 66.3, 22.3 ppm; HRMS (ESI-MS) calcd. for C_11_H_12_N_2_O_2_ [M + H]^+^ = 205.0899. Found: 205.0972. The NMR was consistent with previously reported data [18]. The NMR was measured with a Bruker DRX700 (700 MHz).

*1-(3-Phenylquinoxalin-2-yl)ethane-1,2-diol* (**Quinoxaline-Ph-DPD**): orange solid, *R_f_* = 0.48 (CHCl_3_/MeOH 9:1). ^1^H-NMR (700 MHz, CDCl_3_) δ 8.18 (dd, *J* = 6.4 Hz, *J* = 3.3 Hz, 1H), 8.13 (dd, *J* = 6.1 Hz, *J* = 3.6 Hz, 1H), 7.82 (dd, *J* = 6.4 Hz, *J* = 3.4 Hz, 2H), 7.68 (dd, *J* = 7.8 Hz, *J* = 1.3 Hz, 2H), 7.57–7.53 (m, 3H), 5.30 (dd, *J* = 4.9 Hz, *J* = 3.6 Hz, 1H), 3.74 (dd, *J* = 11.7 Hz, *J* = 3.4 Hz, 1H), 3.54 (dd, *J* = 11.7 Hz, 5.1 Hz, 1H) ppm; ^13^C-NMR (176 MHz, CDCl_3_) δ 153.7, 152.5, 141.8, 139.6, 137.6, 130.5, 130.4, 129.6, 129.4, 129.0, 128.8, 128.4, 70.6, 65.7 ppm. HRMS (ESI-MS) calcd. for C16H14N2O2 [M + H]^+^ = 267.1055. Found: 267.1129. The NMR was consistent with previously reported data [57]. The NMR was measured with a Bruker DRX700 (700 MHz).

### 3.3. General Procedures for the Synthesis of 1,4- and 1,5-Disubstituted Triazoles DPD-Derivatives (Series I and II)

**Synthesis of 1-[(*t*-butyldimethylsilyl)oxy]but-3-yn-2-ol (14)**: to a stirred solution of (*t*-butyldimethylsilyloxy)acetaldehyde (1.0 eq) in dry THF, ethynylmagnesium bromide (0.50 M in THF, 1.3 eq) was added over 15 minutes at 0 °C. After the addition, the reaction was allowed to reach room temperature and stirred for 3 hours. The solvent was removed under reduced pressure, the residue was poured into a cold saturated solution of NH_4_Cl and extracted three times with Et_2_O. The organic layer was washed twice with water and once with brine, dried over MgSO_4_, filtered and concentrated in vacuo to yield **14** as a yellow oil, 99%, *R_f_* = 0.55 (CyH/EtOAc 3:1). ^1^H-NMR (300 MHz, CDCl_3_) δ 4.40–4.37 (m, 1H), 3.79 (dd, *J* = 3.8 Hz, *J* = 10.1 Hz, 1H), 3.66 (dd, *J* = 6.8 Hz, *J* = 10.0 Hz, 1H), 2.62 (d, *J* = 5.1 Hz, 1H), 2.42 (d, *J* = 2.2 Hz, 1H), 0.91 (s, 9H), 0.10 (d, *J* = 1.5 Hz, 6H) ppm; ^13^C-NMR (100 MHz, CDCl_3_) δ 81.9, 73.4, 66.8, 62.9, 25.8, 18.3, −5.4 ppm [58].

**Synthesis of but-3-yne-1,2-diol (15):** to a stirred solution of **14** in MeOH, a Dowex50WX8 100–200 mesh (100 mg/1 mL) was added. The reaction was stirred at room temperature overnight. The mixture was filtered through paper and the solvent was evaporated under reduced pressure to yield **15** as an orange oil, 99%, *R_f_* = 0.50 (CHCl_3_/MeOH 9:1). ^1^H-NMR (300 MHz, CDCl_3_) δ 4.49–4.45 (m, 1H), 3.80–3.68 (m, 1H), 2.51 (d, *J* = 2.2 Hz, 1H), 2.31 (s, 2H) ppm; ^13^C-NMR (100 MHz, CDCl_3_) δ 81.5, 74.3, 66.3, 63.0 ppm [59].

**Synthesis of 2-ethynyl-1,4-dioxaspiro[4.5]decane (16)**: to **15** (1.0 eq) cyclohexanone dimethyl ketal (10.0 eq) and a catalytic amount of *p*-TSA were added. The reaction was stirred at room temperature overnight. The solvent was removed under reduced pressure and the crude was re-dissolved in Et_2_O and washed three times with NaHCO_3_. The organic layer was dried over MgSO_4_, filtered and concentrated in vacuo to yield **16** as a yellow oil, 57%, *R_f_* = 0.42 (CyH/EtOAc 9:1). ^1^H-NMR (300 MHz, CDCl_3_) δ 4.71 (dt, *J* = 2.0 Hz, *J* = 6.3 Hz, 1H), 4.16 (dd, *J* = 6.4 Hz, *J* = 8.0 Hz, 1H), 3.94 (dd, *J* = 6.3 Hz, *J* = 8.0 Hz, 1H), 2.48 (d, *J* = 2.0 Hz, 1H), 1.77–1.72 (m, 2H), 1.65–1.59 (m, 6H), 1.42–1.39 (m, 2H) ppm; ^13^C-NMR (100 MHz, CDCl_3_) δ 111.2, 81.6, 73.7, 69.5, 64.9, 35.6, 25.0, 23.8 ppm [30].

**General procedure for the synthesis of 17a–f:** to a stirred suspension of NaN_3_ (1.5 eq) in DMSO (5 mL), the corresponding bromo compound (1.0 eq) was added. The reaction was stirred at room temperature overnight. The mixture was diluted with diethyl ether and extracted five times with water and once with brine, dried over MgSO_4_, filtered and concentrated in vacuo to yield the desired azide as a colorless/yellowish oil.

**General procedure for the synthesis of 18a–f:** to a stirred solution of **15** (1.0 eq) in a 1:1 mixture of H_2_O/*t*-BuOH, the corresponding azide (1.0 eq), sodium ascorbate (0.5 eq) and CuSO_4_·5H_2_O (5% mol) were added. The reaction was stirred at room temperature overnight. The solvent was evaporated under reduced pressure, the crude was redissolved in ACN (1 mL), filtered and purified by preparative HPLC [49].

*1-[1-(2-Phenylethyl)-1H-1,2,3-triazol-4-yl]ethane-1,2-diol* (**18a**): orange oil, 89%, *R_f_* = 0.24 (CHCl_3_/MeOH 9:1), UHPLC-ESI-MS: *R_t_* = 1.80, *m*/*z* = 234.2 [M + H]^+^. ^1^H-NMR (300 MHz, CD_3_CN) δ 7.56 (s, 1H), 7.31–7.20 (m, 3H), 7.15 (d, *J* = 6.7 Hz, 2H), 4.75 (dd, *J* = 4.3 Hz, *J* = 6.7 Hz, 1H), 4.57 (t, *J* = 7.3 Hz, 2H), 3.72 (dd, *J* = 4.2 Hz, *J* = 11.2 Hz, 1H), 3.60 (dd, *J* = 6.9 Hz, *J* = 11.2 Hz, 1H), 3.17 (t, *J* = 7.2 Hz, 2H), 2.23 (s br, 1H) ppm; ^13^C-NMR (100 MHz, CD_3_CN) δ 149.4, 138.8, 129.7, 129.4, 127.6, 122.8, 68.5, 66.8, 51.9, 36.9 ppm.

*1-(1-Benzyl-1H-1,2,3-triazol-4-yl)ethane-1,2-diol* (**18b**): yellowish oil, 60%, *R_f_* = 0.24 (CHCl_3_/MeOH 9:1), UHPLC-ESI-MS: *R_t_* = 1.70, *m*/*z* = 220.2 [M + H]^+^. ^1^H-NMR (300 MHz, CD_3_CN) δ 7.72 (s, 1H), 7.38–7.29 (m, 5H), 5.51 (s, 2H), 4.79 (dd, *J* = 4.4 Hz, *J* = 6.3 Hz, 1H), 3.75 (dd, *J* = 4.2 Hz, *J* = 11.2 Hz, 1H), 3.64 (dd, *J* = 6.7 Hz, *J* = 11.2 Hz, 1H) ppm; ^13^C-NMR (100 MHz, CD_3_CN) δ 150.1, 136.9, 129.8, 129.3, 128.9, 123.0, 68.5, 66.7, 54.4 ppm.

*1-{1-[.2-(2-Fluorophenyl)ethyl]-1H-1,2,3-triazol-4-yl}ethane-1,2-diol* (**18c**): colorless oil, 62%, *R_f_* = 0.49 (CHCl_3_/MeOH 9:1), UHPLC-ESI-MS: *R_t_* = 1.82, *m*/*z* = 252.2 [M + H]^+^. ^1^H-NMR (300 MHz, CD_3_CN) δ 7.58 (s, 1H), 7.30–7.23 (m, 1H), 7.14–7.04(m, 3H), 4.75(dd, *J* = 4.3 Hz, *J* = 6.8 Hz, 1H), 4.58 (t, *J* = 7.1 Hz, 2H), 3.71 (dd, *J* = 4.2 Hz, *J* = 11.2 Hz, 1H), 3.59 (dd, *J* = 6.9 Hz, *J* = 11.2 Hz, 1H), 3.21 (t, *J* = 7.1 Hz, 2H), 2.23 (s br, 1H) ppm; ^13^C-NMR (100 MHz, CD_3_CN) δ 162.8 (d, *J* = 243.8 Hz), 150.2, 132.9 (d, *J* = 4.6 Hz), 130.6 (d, *J* = 8.2 Hz), 126.2, 126.0 (d, *J* = 3.5 Hz), 123.6, 116.8 (d, *J* = 22.0 Hz), 69.2, 67.5, 51.2, 31.3 (d, *J* = 2.4 Hz) ppm.

*1-{1-[2-(Pyridin-2-yl)ethyl]-1H-1,2,3-triazol-4-yl}ethane-1,2-diol* (**18d**): yellow oil, 88%, *R_f_* = 0.28 (CHCl_3_/MeOH 9:1), UHPLC-ESI-MS: *R_t_* = 0.38, *m*/*z* = 232.2 [M + H]^+^. ^1^H-NMR (300 MHz, CD_3_CN) δ 8.51 (s, 1H), 7.66–7.59 (m, 2H), 7.21–7.12 (m, 2H), 4.75 (t, *J* = 7.1 Hz, 3H), 3.71 (dd, *J* = 4.1 Hz, *J* = 11.2 Hz, 1H), 3.59 (dd, *J* = 6.8 Hz, *J* = 11.1 Hz, 1H), 3.33 (t, *J* = 7.1 Hz, 2H) ppm; ^13^C-NMR (100 MHz, CD_3_CN) δ 158.5, 150.3, 143.3, 137.5, 124.4, 123.2, 122.8, 68.5, 66.8, 50.0, 38.8 ppm.

*6-[4-(1,2-Dihydroxyethyl)-1H-1,2,3-triazol-1-yl]hexanenitrile* (**18e**): orange oil, 72%, *R_f_* = 0.54 (CHCl_3_/MeOH 9:1), UHPLC-ESI-MS: *R_t_* = 1.39, *m*/*z* = 225.2 [M + H]^+^. ^1^H-NMR (300 MHz, CD_3_CN) δ 7.68 (s, 1H), 4.78 (s, 1H), 4.34 (dt, *J* = 1.9 Hz, *J* = 7.1 Hz, 2H), 3.76–3.73 (m, 1H), 3.66–3.57 (m, 1H), 3.08 (s br, 1H), 2.37 (dt, *J* = 1.9 Hz, *J* = 7.1 Hz, 2H), 1.88–1.83 (m, 2H), 1.68–1.58 (m, 2H), 1.44–1.34 (m, 2H) ppm; ^13^C-NMR (100 MHz, CD_3_CN) δ 148.4, 121.5, 119.8, 67.3, 65.6, 49.2, 28.8, 24.9, 24.2, 16.0 ppm.

*1-[1-(2-Cyclohexylethyl)-1H-1,2,3-triazol-4-yl]ethane-1,2-diol* (**18f**): orange oil, 73%, *R_f_* = 0.43 (CHCl_3_/MeOH 9:1), UHPLC-ESI-MS: *R_t_* = 2.20, *m*/*z* = 240.2 [M + H]^+^. ^1^H-NMR (300 MHz, CD_3_CN) δ 7.69 (s, 1H), 4.78 (t, *J* = 5.3 Hz, 1H), 4.35 (t, *J* = 7.5 Hz, 2H), 3.74 (s, 1H), 3.67–3.62 (m, 1H), 2.21 (s br, 1H), 1.78–1.63 (m, 7H), 1.24–1.17 (m, 4H), 1.0–0.94 (m, 2H) ppm; ^13^C-NMR (100 MHz, CD_3_CN) δ 150.4, 123.5, 69.2, 67.5, 49.4, 39.0, 36.4, 34.2, 27.8, 27.5 ppm.

**Synthesis of 1-[1-(2-phenylethyl)-1*H*-1,2,3-triazol-5-yl]ethane-1,2-diol (19a)**: to a stirred solution of **15** (1.0 eq) in 1,4-dioxane, (2-azidoethyl)benzene (**17a**) (1.0 eq) and Cp*RuCl(PPh_3_)_2_ (2% mol) were added. The reaction was stirred at reflux overnight. The solvent was evaporated under reduced pressure, the crude was re-dissolved in ACN (1 mL), filtered and purified by preparative HPLC to yield **19a** as a yellow solid, 87%, *R_f_* = 0.17 (CHCl_3_/MeOH 9:1), UHPLC-ESI-MS: *R_t_* = 1.75, *m*/*z* = 234.2 [M + H] ^+^. ^1^H-NMR (300 MHz, CD_3_CN) δ 7.51 (s, 1H), 7.28 (t, *J* = 7.3 Hz, 2H), 7.23 (t, *J* = 7.3 Hz, 1H), 7.15 (d, *J* = 7.1 Hz, 2H), 4.62 (d, *J* = 6.3 Hz, 1H), 4.61–4.58 (m, 2H), 3.70 (s br, 1H), 3.62 (dd, *J* = 6.6 Hz, *J* = 11.3 Hz, 1H), 3.56 (dd, *J* = 4.9 Hz, *J* = 11.3 Hz, 1H), 3.21 (t, *J* = 7.4 Hz, 2H), 2.21 (s, 1H) ppm; ^13^C-NMR (100 MHz, CD_3_CN) δ 139.0, 138.5, 132.1, 129.7, 129.4, 127.6, 65.5 (d, *J* = 7.6 Hz), 50.4, 37.0 ppm.

**Synthesis of 4-{1,4-dioxaspiro[4.5]decan-2-yl}-1*H*-1,2,3-triazole (20)**: to a stirred solution of **16** (1.0 eq) in a 1:1 mixture of H_2_O/*t*-BuOH, trimethylsilyl azide (10.0 eq), sodium ascorbate (0.5 eq) and CuSO_4_·5H_2_O (5% mol) were added. The reaction was stirred at room temperature overnight. The solvent was evaporated under reduced pressure, the crude was re-dissolved in EtOAc and extracted three times with water. The organic layer was dried over MgSO_4_, filtered and concentrated in vacuo. The crude was purified using CyH/TBME (3:1) as an eluent to yield 20 as a yellowish oil, 36%, *R_f_* = 0.61 (CHCl_3_/MeOH 9:1), UHPLC-ESI-MS: *R_t_* = 2.13, *m*/*z* = 210.2 [M + H] ^+^. ^1^H-NMR (300 MHz, CD_3_CN) δ 7.72 (s, 1H), 5.25 (t, *J* = 6.6 Hz, 1H), 4.31–4.26 (m, 1H), 4.00–3.95 (m, 1H), 2.37 (s br, 1H), 1.64–1.58 (m, 8H), 1.43–1.40 (m, 2H) ppm; ^13^C-NMR (100 MHz, CD_3_CN) δ 147.4, 130.0, 111.1, 70.9, 69.8, 36.8, 35.9, 25.8, 24.7, 24.6 ppm.

**General procedure for the synthesis of 21h–k and 22h–k:** to a stirred solution of **20** (1.0 eq) in dry THF, K_2_CO_3_ (2.0 eq) and the corresponding alkyl halide (bromide or iodide) were added. The reaction was stirred at reflux overnight. The solvent was evaporated under reduced pressure, the crude was re-dissolved in ACN (1 mL), filtered and purified by preparative HPLC.

For each compound, two different fractions were isolated corresponding to the 1,4- and 1,5-disubstitued products. The different substitution was determined by HMBC of two representative samples (**21i**, **22i**).

**General procedure for the synthesis of 18g–k and 19h–k:** a stirred solution of **20** (or **21h**–**k**, or **22h**–**k**) in 1,4-dioxane was cooled to 0 °C using an ice bath. A catalytic amount of 12 M HCl was added. The reaction was stirred at room temperature overnight. The solvent was evaporated under reduced pressure; the crude was re-dissolved in Et_2_O and extracted with water. The aqueous layer was extracted three times with Et_2_O and dried in vacuo to yield the corresponding products **18g**–**k** and **19h**–**k**.

*1-(1H-1,2,3-Triazol-4-yl)ethane-1,2-diol* (**18g**): yellowish oil, 98%, *R_f_* = 0.13 (CHCl_3_/MeOH 9:1), UHPLC-ESI-MS: *R_t_* = 0.32, *m*/*z* = 130.3 [M + H]^+^. ^1^H-NMR (300 MHz, MeOD) δ 8.38 (s, 1H), 5.05 (t, *J* = 5.5 Hz, 1H), 3.86–3.74 (m, 2H) ppm; ^13^C-NMR (100 MHz, MeOD) δ 146.0, 126.4, 67.0, 66.2 ppm.

*1-(1-Methyl-1H-1,2,3-triazol-4-yl)ethane-1,2-diol* (**18h**): colorless oil, 66%, *R_f_* = 0.29 (CHCl_3_/MeOH 9:1), UHPLC-ESI-MS: *R_t_* = 0.45, *m*/*z* = 144.2 [M + H]^+^. ^1^H-NMR (300 MHz, MeOD) δ 8.40 (s, 1H), 5.04 (t, *J* = 5.5 Hz, 1H), 4.35 (s, 3H), 3.94–3.80 (m, 2H) ppm; ^13^C-NMR (100 MHz, MeOD) δ 151.9, 133.5, 70.0, 68.2, 42.9 ppm.

*1-(1-Methyl-1H-1,2,3-triazol-5-yl)ethane-1,2-diol* (**19h**): colorless oil, 65%, *R_f_* = 0.32 (CHCl_3_/MeOH 9:1), UHPLC-ESI-MS: *R_t_* = 0.65, *m*/*z* = 144.1 [M + H]^+^. ^1^H-NMR (300 MHz, MeOD) δ 7.60 (s, 1H), 4.81 (dd, *J* = 4.8 Hz, *J* = 6.8 Hz, 1H), 4.13 (s, 3H), 3.81–3.68 (m, 2H) ppm; ^13^C-NMR (100 MHz, MeOD) δ 150.6, 133.4, 68.7, 67.0, 41.7 ppm.

*1-[1-(Cyclopropylmethyl)-1H-1,2,3-triazol-4-yl]ethane-1,2-diol* (**18i**): colorless oil, 73%, *R_f_* = 0.32 (CHCl_3_/MeOH 9:1), UHPLC-ESI-MS: *R_t_* = 1.25, *m*/*z* = 184.2 [M + H]^+^. ^1^H-NMR (300 MHz, CD_3_CN) δ 8.29 (d, *J* = 9.1 Hz, 1H), 6.21 (s br, 2H), 5.02 (dd, *J* = 3.9 Hz, *J* = 6.4 Hz, 1H), 4.36 (dd, *J* = 2.5 Hz, *J* = 7.4 Hz, 2H), 3.80 (d, *J* = 5.3 Hz, 2H), 1.40–1.35 (m, 1H), 0.70–0.64 (m, 2H), 0.52–0.47 (m, 2H) ppm; ^13^C-NMR (100 MHz, CD_3_CN) δ 146.8, 125.8, 66.9, 65.7, 57.8, 11.1, 4.5 ppm.

*1-[1-(Cyclopropylmethyl)-1H-1,2,3-triazol-5-yl]ethane-1,2-diol* (**19i**): colorless oil, 77%, *R_f_* = 0.35 (CHCl_3_/MeOH 9:1), UHPLC-ESI-MS: *R_t_* = 1.31, *m*/*z* = 184.2 [M + H]^+^. ^1^H-NMR (300 MHz, CD_3_CN) δ 7.87 (s, 1H), 4.91 (t, *J* = 5.5 Hz, 1H), 4.34 (d, *J* = 7.3 Hz, 2H), 3.76 (d, *J* = 5.5 Hz, 2H), 1.43–1.38 (m, 1H), 0.64–0.58 (m, 2H), 0.49–0.46 (m, 2H) ppm; ^13^C-NMR (100 MHz, CD_3_CN) δ 140.3, 130.7, 65.5, 65.4, 55.2, 11.7, 4.6 ppm.

*1-(1-Butyl-1H-1,2,3-triazol-4-yl)ethane-1,2-diol* (**18j**): colorless oil, 85%, *R_f_* = 0.37 (CHCl_3_/MeOH 9:1), UHPLC-ESI-MS: *R_t_* = 1.45, *m*/*z* = 186.2 [M + H]^+^. ^1^H-NMR (300 MHz, MeOD) δ 8.28 (s, 1H), 4.77 (t, *J* = 5.5 Hz, 1H), 4.39 (t, *J* = 7.2 Hz, 2H), 3.63–3.49 (m, 2H), 1.81–1.71(m, 2H), 1.23–1.11 (m, 2H), 0.76 (t, *J* = 7.4 Hz, 3H) ppm; ^13^C-NMR (100 MHz, MeOD) δ 147.5, 126.7, 67.2, 66.1, 53.8, 32.6, 20.5, 13.7 ppm.

*1-(1-Butyl-1H-1,2,3-triazol-5-yl)ethane-1,2-diol* (**19j**): colorless oil, 95%, *R_f_* = 0.39 (CHCl_3_/MeOH 9:1), UHPLC-ESI-MS: *R_t_* = 1.50, *m*/*z* = 186.2 [M + H]^+^. ^1^H-NMR (300 MHz, MeOD) δ 8.21 (s, 1H), 4.98 (t, *J* = 5.7 Hz, 1H), 4.62 (dd, *J* = 6.5 Hz, *J* = 8.4 Hz, 2H), 3.91–3.78 (m, 2H), 2.04–1.94 (m, 2H), 1.49–1.37 (m, 2H), 1.00 (t, *J* = 7.3 Hz, 3H) ppm; ^13^C-NMR (100 MHz, MeOD) δ 142.7, 130.1, 65.9, 65.8, 51.5, 32.8, 20.7, 13.8 ppm.

*1-[1-(2-Ethoxyethyl)-1H-1,2,3-triazol-4-yl]ethane-1,2-diol* (**18k**): colorless oil, 91%, *R_f_* = 0.31 (CHCl_3_/MeOH 9:1), UHPLC-ESI-MS: *R_t_* = 1.21, *m*/*z* = 202.2 [M + H]^+^. ^1^H-NMR (300 MHz, CD_3_CN) δ 7.92 (s, 1H), 4.89 (t, *J* = 5.1 Hz, 1H), 4.54 (t, *J* = 5.1 Hz, 2H), 3.83–3.68 (m, 4H), 3.47 (q, *J* = 7.0 Hz, 2H), 1.10 (t, *J* = 7.0 Hz, 3H) ppm; ^13^C-NMR (100 MHz, CD_3_CN) δ 148.3, 124.9, 68.8, 67.7, 66.9, 66.3, 52.1, 15.2 ppm.

*1-[1-(2-Ethoxyethyl)-1H-1,2,3-triazol-4-yl]ethane-1,2-diol* (**19k**): colorless oil, 82%, *R_f_* = 0.37 (CHCl_3_/MeOH 9:1), UHPLC-ESI-MS: *R_t_* = 1.23, *m*/*z* = 202.2 [M + H]^+^. ^1^H-NMR (300 MHz, MeOD) δ 8.10 (s, 1H), 5.00 (t, *J* = 5.5 Hz, 1H), 4.74 (dd, *J* = 3.7 Hz, *J* = 5.3 Hz, 2H), 3.84 (t, *J* = 5.2 Hz, 2H), 3.77 (t, *J* = 5.8 Hz, 2H), 3.46–3.38 (m, 2H), 1.05 (t, *J* = 7.0 Hz, 3H) ppm; ^13^C-NMR (100 MHz, MeOD) δ 143.4, 130.3, 69.6, 67.7, 66.0, 65.9, 51.7, 15.3 ppm.

### 3.4. General Procedures for the Synthesis of 3,5-Disubstituted Isoxazoles DPD Derivatives (Series III and IV)

**General procedure for the synthesis of 24l–r:** to a stirred solution of the corresponding aldehyde (1.0 eq) in EtOH (10 mL), Et_3_N (1.5 eq) and NH_2_OH*HCl (1.5 eq) dissolved in water (10 mL) were added. The reaction was stirred at room temperature for 1–3 hours (monitored by TLC). The solvent was evaporated under reduced pressure; the crude was re-dissolved in EtOAc and extracted three times with water. The organic layer was dried over MgSO_4_, filtered and concentrated in vacuo to yield the corresponding oxime. All the resulting compounds were used in the next step without being purified.

**General procedure for the synthesis of 25l–r:** to a stirred solution of the corresponding oxime (1.0 eq) in DMF, *N*-chlorosuccinimide (1.0 eq) was added in two portions. The reaction was stirred at room temperature for 1–2 h (monitored by TLC). The crude was diluted with Et_2_O and extracted five times with water and once with brine. The organic layer was dried over MgSO_4_, filtered and concentrated in vacuo to yield the corresponding chloro-oxime. All the resulting compounds were used in the next step without being purified.

**General procedure for the synthesis of 26l–r:** to a stirred solution of **15** (1.0 eq) in a 1:1 mixture of H_2_O/*t*-BuOH, the corresponding chloro-oxime (1.0 eq), sodium ascorbate (0.5 eq), CuSO_4_·5H_2_O (5% mol) and KHCO_3_ (4.3 eq) were added. The mixture was stirred at room temperature overnight. The solvent was evaporated under reduced pressure; the crude was redissolved in ACN (1 mL), filtered and purified by preparative HPLC [49].

*1-[3-(4-Methylphenyl)-1,2-oxazol-5-yl]ethane-1,2-diol* (**26l**): white solid, 82%, *R_f_* = 0.38 (CHCl_3_/MeOH 9:1), UHPLC-ESI-MS: *R_t_* = 2.15, *m*/*z* = 220.1 [M + H]^+^. ^1^H-NMR (300 MHz, CD_3_CN) δ 7.73 (d, *J* = 8.0 Hz, 2H), 7.31 (d, *J* = 7.9 Hz, 2H), 6.67 (s, 1H), 4.82 (d, *J* = 5.2 Hz, 1H), 3.92 (s br, 1H), 3.83–3.71 (m, 2H), 3.14 (s br, 1H), 2.38 (s, 3H) ppm; ^13^C-NMR (100 MHz, CD_3_CN) δ 175.3, 163.7, 142.0, 131.3, 128.2, 127.9, 101.2, 69.2, 66.0, 22.0 ppm.

*1-[3-(3-Chlorophenyl)-1,2-oxazol-5-yl]ethane-1,2-diol* (**26m**): white solid, 87%, *R_f_* = 0.51 (CHCl_3_/MeOH 9:1), UHPLC-ESI-MS: *R_t_* = 2.24, *m*/*z* = 240.0 [M + H]^+^. ^1^H-NMR (300 MHz, CD_3_CN) δ 7.87 (s, 1H), 7.77 (dd, *J* = 5.4 Hz, *J* = 6.8 Hz, 1H), 7.48 (d, *J* = 5.8 Hz, 2H), 6.74 (s, 1H), 4.84 (t, *J* = 5.3 Hz, 1H), 3.78 (dq, *J* = 5.3 Hz, *J* = 11.4 Hz, 2H) ppm; ^13^C-NMR (100 MHz, CD_3_CN) δ 175.2, 162.0, 135.4, 132.0, 131.6, 130.9, 127.5, 126.1, 100.8, 68.5, 65.2 ppm.

*1-[3-(2,4-Difluorophenyl)-1,2-oxazol-5-yl]ethane-1,2-diol* (**26n**): white solid, 78%, *R_f_* = 0.49 (CHCl_3_/MeOH 9:1), UHPLC-ESI-MS: *R_t_* = 2.06, *m*/*z* = 242.2 [M + H]^+^. ^1^H-NMR (300 MHz, CD_3_CN) δ 7.97–7.89 (m, 1H), 7.14–7.06 (m, 2H), 6.69 (d, *J* = 3.3 Hz, 1H), 4.86 (t, *J* = 5.3 Hz, 1H), 3.85–3.73 (m, 2H) ppm; ^13^C-NMR (100 MHz, CD_3_CN) δ 174.8, 164.9 (dd, *J* = 8.5 Hz, *J* = 246.4 Hz), 161.4 (dd, *J* = 8.5 Hz, *J* = 249.4 Hz), 157.9, 131.6 (dd, *J* = 4.6 Hz, *J* = 10.1 Hz), 114.7 (dd, *J* = 3.9 Hz, *J* = 12.6 Hz), 113.2 (dd, *J* = 3.6 Hz, *J* = 21.9 Hz), 105.6 (t, *J* = 26.1 Hz), 102.7 (d, *J* = 7.4 Hz), 68.5, 65.3 ppm.

*1-[3-(Pyridin-3-yl)-1,2-oxazol-5-yl]ethane-1,2-diol* (**26o**): orange solid, 71%, *R_f_* = 0.22 (CHCl_3_/MeOH 9:1), UHPLC-ESI-MS: *R_t_* = 1.14, *m*/*z* = 207.2 [M + H]^+^. ^1^H-NMR (300 MHz, CD_3_CN) δ 9.04 (s, 1H), 8.67 (s, 1H), 8.18 (d, *J* = 7.9 Hz, 1H), 7.46 (dd, *J* = 5.3 Hz, *J* = 7.2 Hz, 1H), 6.79 (s, 1H), 4.87 (t, *J* = 5.3 Hz, 1H), 3.85–3.73 (m, 2H) ppm; ^13^C-NMR (100 MHz, CD_3_CN) δ 175.4, 160.9, 151.9, 148.7, 135.0, 126.2, 124.9, 100.7, 68.5, 65.3 ppm.

*1-(3-Cyclopropyl-1,2-oxazol-5-yl)ethane-1,2-diol* (**26p**): yellow oil, 63%, *R_f_* = 0.12 (CHCl_3_/MeOH 9:1), UHPLC-ESI-MS: *R_t_* = 1.91, *m*/*z* = 170.2 [M + H]^+^. ^1^H-NMR (300 MHz, CD_3_CN) δ 5.98 (s, 1H), 4.71 (ddd, *J* = 0.6 Hz, *J* = 4.6 Hz, *J* = 6.1 Hz, 1H), 3.74–3.62 (m, 2H), 2.00–1.91 (m, 1H), 1.03–0.97 (m, 2H), 0.78–0.72 (m, 2H) ppm; ^13^C-NMR (100 MHz, CD_3_CN) δ 173.5, 167.3, 99.7, 68.3, 65.2, 8.3, 7.8 ppm.

*1-[3-(Oxolan-3-yl)-1,2-oxazol-5-yl]ethane-1,2-diol* (**26q**): yellow oil, 77%, *R_f_* = 0.50 (CHCl_3_/MeOH 9:1), UHPLC-ESI-MS: *R_t_* = 1.53, *m*/*z* = 200.2 [M + H]^+^. ^1^H-NMR (300 MHz, CD_3_CN) δ 6.22 (s, 1H), 4.77–4.73 (m, 1H), 4.03–3.98 (m, 1H), 3.94–3.85 (m, 1H), 3.82–3.76 (m, 1H), 3.74–3.65 (m, 2H), 3.55–3.47 (m, 1H), 2.37–2.25 (m, 2H), 2.09–2.00 (m, 1H) ppm; ^13^C-NMR (100 MHz, CD_3_CN) δ 174.1, 166.1, 101.2, 72.7, 68.5, 65.3, 63.7, 37.4, 32.5 ppm.

*1-(3-Cyclohexyl-1,2-oxazol-5-yl)ethane-1,2-diol* (**26r**): yellow oil, 89%, *R_f_* = 0.31 (CHCl_3_/MeOH 9:1), UHPLC-ESI-MS: *R_t_* = 2.13, *m*/*z* = 212.2 [M + H]^+^. ^1^H-NMR (300 MHz, CD_3_CN) δ 6.18 (s, 1H), 4.73 (t, *J* = 5.4 Hz, 1H), 3.71 (dq, *J* = 5.4 Hz, *J* = 11.3 Hz, 2H), 2.70 (dt, *J* = 3.3 Hz, *J* = 10.7 Hz, 1H), 2.21 (s br, 2H), 1.90–1.69 (m, 5H), 1.50–1.25 (m, 5H) ppm; ^13^C-NMR (100 MHz, CD_3_CN) δ 173.9, 169.8, 101.5, 69.2, 66.0, 37.3, 33.4, 27.3, 27.2 ppm.

**Synthesis of ethyl (2*E*)-2-(hydroxyimino)acetate (27):** to a stirred solution of ethyl glyoxalate (50% solution in toluene, 1.0 eq) in EtOH, Et_3_N (1.5 eq) and NH_2_OH·HCl (1.5 eq) dissolved in water (10 mL) were added. The reaction was stirred at room temperature for 2 hours (monitored by TLC). The solvent was evaporated under reduced pressure; the crude was re-dissolved in Et_2_O and extracted three times with water. The organic layer was dried over MgSO_4_, filtered and concentrated in vacuo to yield **27** as a colorless oil, 84%, *R_f_* = 0.64 (CHCl_3_/MeOH 9:1). ^1^H-NMR (300 MHz, CDCl_3_) δ 9.83 (s br, 1H), 7.56 (s, 1H), 4.32 (q, *J* = 7.1 Hz, 2H), 1.34 (t, *J* = 7.1 Hz, 3H) ppm; ^13^C-NMR (100 MHz, CDCl_3_) δ 162.4, 141.6, 61.8, 13.8 ppm [60].

**Synthesis of ethyl 5-{1,4-dioxaspiro[4.5]decan-2-yl}-1,2-oxazole-3-carboxylate (28):** to a stirred solution of **16** (1.0 eq) in THF, **27** (2.0 eq) and NaOCl (40.0 eq portion wise over 12 hours) were added. The reaction was stirred at room temperature for 12 hours. The solvent was evaporated under reduced pressure, the crude was re-dissolved in DCM and washed three times with water. The organic layer was dried over MgSO_4_, filtered and concentrated in vacuo. The crude was re-dissolved in ACN (1 mL), filtered and purified by preparative HPLC to yield **28** as a yellowish oil, 36%, *R_f_* = 0.57 (CyH/EtOAC 3:1), UHPLC-ESI-MS: *R_t_* = 3.04, *m*/*z* = 282.2 [M + H]^+^. ^1^H-NMR (300 MHz, CDCl_3_) δ 6.67 (s, 1H), 5.23 (t, *J* = 6.0 Hz, 1H), 4.43 (q, *J* = 7.2 Hz, 2H), 4.35 (dd, *J* = 6.7 Hz, *J* = 8.6 Hz, 1H), 4.09 (dd, *J* = 5.4 Hz, *J* = 8.6 Hz, 1H), 1.71–1.62 (m, 9H), 1.41 (t, *J* = 7.1 Hz, 4H) ppm; ^13^C-NMR (100 MHz, CDCl_3_) δ 173.5, 159.8, 156.3, 111.9, 102.5, 69.8, 68.1, 62.2, 35.8, 34.9, 25.0, 23.9, 23.8, 14.1 ppm.

**Synthesis of 5-{1,4-dioxaspiro[4.5]decan-2-yl}-1,2-oxazole-3-carboxylic acid (30)**: a stirred solution of **28** in THF was cooled to 0 °C using an ice bath. A solution of 10 M NaOH (5.0 eq) was added dropwise and the reaction was stirred at room temperature overnight. The solvent was evaporated under reduced pressure; the crude was re-dissolved in DCM and extracted with water. The aqueous layer was acidified with 1M HCl until pH = 1 and extracted three times with CHCl_3_/*i*-PrOH (7:3). The organic layer was dried over MgSO_4_, filtered and concentrated in vacuo to yield **30** as a white solid, 99%, *R_f_* = 0.17(CHCl_3_/MeOH 5:1), UHPLC-ESI-MS: *R_t_* = 2.37, *m*/*z* = 254.2 [M + H] ^+^. ^1^H-NMR (300 MHz, CDCl_3_) δ 6.74 (s, 1H), 5.26 (t, *J* = 5.9 Hz, 1H), 4.38 (dd, *J* = 6.6 Hz, *J* = 8.7 Hz, 1H), 4.12 (dd, *J* = 5.3 Hz, *J* = 8.7 Hz, 1H), 1.73–1.60 (m, 8H), 1.47–1.43 (m, 2H) ppm; ^13^C-NMR (100 MHz, CDCl_3_) δ 174.2, 162.4, 155.6, 112.1, 102.8, 69.8, 68.1, 35.9, 34.9, 24.9, 23.9, 23.8 ppm.

**General procedure for the synthesis of 32b, 32s–z:** the reactions were performed in parallel in 15 mL reaction tubes in a 24 position Mettler-Toledo Miniblock® equipped with a heat transfer block and inert gas manifold. Each reaction tube was loaded with a previously prepared solution of 30 mg of **28** (1.0 eq) in 2 mL of DMF, DIPEA (5.0 eq), HOBt (2.0 eq), EDC·HCl (2.5 eq). Then the corresponding amine was added (2.0 eq). The reaction mixtures were stirred at room temperature overnight. The reaction conversion was confirmed through a UHPLC check of some representative samples. The mixtures were evaporated until dryness. The crudes were re-dissolved in 1.0 mL of ACN, filtered and purified with preparative HPLC (gradient acetonitrile/water with 0.1% formic acid, 2–98%).

**General procedure for the synthesis of 29, 31, 33b, 33s–z:** a stirred solution of **28** (or **30**, or **32b**, or **32s**–**z**) was cooled to 0 °C using an ice bath. A catalytic amount of concentrated HCl was added. The reactions were stirred at room temperature overnight. The solvent was evaporated under reduced pressure, the crudes were re-dissolved in ACN (1 mL), filtered and purified by preparative HPLC.

*Ethyl 5-(1,2-dihydroxyethyl)-1,2-oxazole-3-carboxylate* (**29**): colorless oil, 46%, *R_f_* = 0.44 (CHCl_3_/MeOH 9:1), UHPLC-ESI-MS: *R_t_* = 1.51, *m*/*z* = 202.2 [M + H]^+^. ^1^H-NMR (300 MHz, CD_3_CN) δ 6.65 (s, 1H), 4.84 (t, *J* = 5.3 Hz, 1H), 4.37 (q, *J* = 7.1 Hz, 2H), 3.76 (dd, *J* = 4.2 Hz, *J* = 5.2 Hz, 2H), 2.18 (s br, 1H), 1.35 (t, *J* = 7.1 Hz, 3H) ppm; ^13^C-NMR (100 MHz, CD_3_CN) δ 176.2, 160.8, 157.4, 103.0, 68.3, 65.1, 62.9, 14.3 ppm.

*5-(1,2-Dihydroxyethyl)-1,2-oxazole-3-carboxylic acid* (**31**): colorless oil, 55%, *R_f_* = 0.11 (CHCl_3_/MeOH 9:1), UHPLC-ESI-MS: *R_t_* = 0.42, *m*/*z* = 174.2 [M + H]^+^. ^1^H-NMR (300 MHz, CD_3_CN) δ 6.65 (s, 1H), 4.84 (t, *J* = 5.1 Hz, 1H), 3.81–3.70 (m, 2H) ppm; ^13^C-NMR (100 MHz, CD_3_CN) δ 176.2, 161.0, 157.2, 103.2, 68.3, 65.1 ppm.

*N-Benzyl-5-(1,2-dihydroxyethyl)-1,2-oxazole-3-carboxamide* (**33b**): white solid, 58%, *R_f_* = 0.25 (CHCl_3_/MeOH 9:1), UHPLC-ESI-MS: *R_t_* = 1.89, *m*/*z* = 263.2 [M + H]^+^. ^1^H-NMR (300 MHz, Acetone-*d*_6_) δ 7.40–7.27 (m, 4H), 7.26–7.22 (m, 1H), 6.68 (s, 1H), 4.91 (t, *J* = 5.4 Hz, 1H), 4.59 (s, 2H), 3.89–3.78 (m, 2H) ppm; ^13^C-NMR (100 MHz, Acetone-*d*_6_) δ 177.2, 160.6, 141.0, 137.3, 130.2, 129.4, 128.9, 102.9, 69.5, 66.4, 44.4 ppm.

*5-(1,2-Dihydroxyethyl)-N-(4-fluorophenyl)-1,2-oxazole-3-carboxamide* (**33s**): white solid, 58%, *R_f_* = 0.25 (CHCl_3_/MeOH 9:1), UHPLC-ESI-MS: *R_t_* = 1.97, *m*/*z* = 267.2 [M + H]^+^. ^1^H-NMR (300 MHz, Acetone-*d*_6_) δ 7.92–7.88 (m, 2H), 7.16 (t, *J* = 8.8 Hz, 2H), 6.76 (s, 1H), 4.95 (t, *J* = 5.4 Hz, 1H), 3.92–3.81 (m, 2H) ppm; ^13^C-NMR (100 MHz, Acetone-*d*_6_) δ 177.6, 162.8, 159.8 (d, *J* = 133.8 Hz), 159.6, 136.3 (d, *J* = 2.7 Hz), 124.1 (d, *J* = 7.7 Hz), 117.2 (d, *J* = 22.6 Hz), 103.1, 69.5, 66.4 ppm.

*5-(1,2-Dihydroxyethyl)-N-[(thiophen-2-yl)methyl]-1,2-oxazole-3-carboxamide* (**33t**): white solid, 65%, *R_f_* = 0.34 (CHCl_3_/MeOH 9:1), UHPLC-ESI-MS: *R_t_* = 1.79, *m*/*z* = 269.2 [M + H]^+^. ^1^H-NMR (300 MHz, Acetone-*d*_6_) δ 8.43 (s br, 0.5H), 7.32 (dd, *J* = 1.3 Hz, *J* = 5.1 Hz, 1H), 7.06 (dd, *J* = 1.1 Hz, *J* = 3.4 Hz, 1H), 6.95 (dd, *J* = 3.5 Hz, *J* = 5.1 Hz, 1H), 6.68 (s, 1H), 4.99 (s br, 0.5H), 4.90 (t, *J* = 5.1 Hz, 1H), 4.77–4.75 (m, 2H), 4.15 (s br, 0.5H), 3.84–3.81 (m, 2H), 2.87 (s br, 0.5H) ppm; ^13^C-NMR (100 MHz, Acetone-*d*_6_) δ 177.3, 160.4, 143.6, 141.4, 128.5, 127.8, 126.8, 102.9, 69.6, 66.5, 39.3 ppm.

*5-(1,2-Dihydroxyethyl)-N-[(pyridin-3-yl)methyl]-1,2-oxazole-3-carboxamide* (**33u**): yellow oil, 37%, *R_f_* = 0.33 (CHCl_3_/MeOH 9:1), UHPLC-ESI-MS: *R_t_* = 0.38, *m*/*z* = 264.2 [M + H]^+^. ^1^H-NMR (300 MHz, MeOD) δ 8.56 (s, 1H), 8.44 (s br, 1H), 7.86 (d, *J* = 7.9 Hz, 1H), 7.43 (dd, *J* = 4.9 Hz, *J* = 7.8 Hz, 1H), 6.70 (s, 1H), 4.84 (d, *J* = 5.8 Hz, 1H), 4.59 (s, 1H), 3.80 (dd, *J* = 3.3 Hz, *J* = 5.6 Hz, 2H) ppm; ^13^C-NMR (100 MHz, MeOD) δ 176.6, 161.6, 159.6, 149.6, 149.0, 137.8, 136.5, 125.3, 102.1, 68.8, 65.6, 41.6 ppm.

*5-(1,2-Dihydroxyethyl)-N-(2-methoxyethyl)-1,2-oxazole-3-carboxamide* (**33v**): yellow oil, 78%, *R_f_* = 0.38 (CHCl_3_/MeOH 9:1), UHPLC-ESI-MS: *R_t_* = 1.23, *m*/*z* = 231.2 [M + H]^+^. ^1^H-NMR (300 MHz, Acetone-*d*_6_) δ 6.64 (s, 1H), 4.90 (t, *J* = 5.4 Hz, 1H), 3.83 (dd, *J* = 3.6 Hz, *J* = 5.4 Hz, 2H), 3.58–3.50 (m, 4H), 3.32 (s, 3H), 2.85 (s br, 2H) ppm; ^13^C-NMR (100 MHz, Acetone-*d*_6_) δ 177.3, 160.5, 147.7, 102.8, 72.4, 69.6, 66.5, 59.6, 40.5 ppm.

*1-[3-(Pyrrolidine-1-carbonyl)-1,2-oxazol-5-yl]ethane-1,2-diol* (**33z**): yellow oil, 79%, *R_f_* = 0.30 (CHCl_3_/MeOH 9:1), UHPLC-ESI-MS: *R_t_* = 1.51, *m*/*z* = 227.2 [M + H] ^+^. ^1^H-NMR (300 MHz, Acetone-*d*_6_) δ 6.58 (s, 1H), 4.90 (t, *J* = 5.3 Hz, 1H), 3.83 (t, *J* = 5.1 Hz, 2H), 3.76 (t, *J* = 6.6 Hz, 2H), 3.54 (t, *J* = 6.6 Hz, 2H), 2.84 (s br, 2H), 1.99–1.89 (m, 4H) ppm; ^13^C-NMR (100 MHz, Acetone-*d*_6_) δ 175.8, 161.7, 149.6, 104.2, 69.6, 66.6, 50.0, 48.2, 27.8, 25.5 ppm.

### 3.5. Biology

All chemicals were purchased from Sigma (Hamburg, Germany) if not otherwise stated. (*S*)-DPD was purchased from OMM Scientific (Dallas, TX, USA). The ATP Bioluminescence kit CLS II and Kinase Glo Luminescence assay kit were respectively purchased from Roche Scientific (Manheim, Germany) and Promega (Madison, WI, USA).

#### 3.5.1. LsrK Overexpression and Purification

*E. coli* MET1158 (*E. coli*, amp resistance, BL21 (DE3) luxS-, with pMET1144 (lsrK-His in pET21b)), kindly donated by Prof. Karina Xavier (Instituto Gulbenkian de Ciência, Portugal) [61], was used for the overexpression of LsrK from *S. typhimurium*. The bacteria were grown overnight in 2 × YPTG (yeast, tryptone, phosphate buffer and glucose) mediums supplemented with 100 µg/mL ampicillin. At the exponential phase, protein expression was induced by the addition of 0.1 mM isopropyl β-D-1 thiogalactopyranoside for 9 h at 22 °C (250 rpm). Cells were harvested and frozen overnight before proceeding with lysis and purification, according to the literature [62].

#### 3.5.2. DPD Activity Evaluation

Phosphorylation of DPD by LsrK was evaluated with a bioluminescence-based assay, ATP Bioluminescence kit CLSII (Roche) as previously described in Reference [61]. DPD was plated at 200 µM and 400 µM and a reaction mixture containing 200 nM Lsrk and 20 µM ATP in assay buffer (25 mM triethanolamine, pH 7.4, 200 µM MgCl_2_). Commercially available DPD was tested for comparison at 200 µM. The level of ATP was monitored by the ATP Bioluminescence kit CLSII following the manufacturer’s instructions. The experiment was performed in the kinetic-mode, monitoring the luminescence every 2 min within a time window of 30 min at the Varioskan LUX plate reader (Thermo Fisher Scientific, Vantaa, Finland).

#### 3.5.3. Screening of DPD-Related Compounds

The activity of DPD-related compounds was evaluated in an LsrK inhibition assay. Compounds were plated in a 384 well-plate to a final concentration of 200 µM in triplicate. A 300 nM LsrK and 300 µM DPD diluted in an assay buffer (25 mM triethanolamine, pH 7.4, 200 µM MgCl_2_, 0.1 mg/mL BSA) were added to the plate followed by 100 µM ATP to start the reaction. After 15 min of reaction, the Kinase Glo Luminescence assay reagent was added according to the manufacturer’s instructions. The experiment was carried on in end-point mode and the luminescence was recorded at the Varioskan LUX plate reader.

## 4. Conclusions

Resistance to antibiotics poses a continuous threat to public health. In the last few decades, receptors able to modulate QS started to be considered interesting targets for anti-infective therapy and the modulation/inhibition of QS has become an appealing strategy against bacterial resistance. Several studies have already shown that interference with QS affects biofilm formation and biofilm properties (e.g., thickness, mass). Particularly, DPD, the key compound in the biosynthesis of AI-2, is able to modulate QS in both Gram-negative and Gram-positive bacteria. Accordingly, DPD-analogs may have great potential as QSI and, therefore, as antimicrobial drugs. Of note, two different DPD-related compounds (i.e. isobutyl-DPD and phenyl-DPD) in combination with gentamicin have almost completely cleared the pre-existing biofilms in *E. coli* and *P. aeruginosa*, respectively [63].

In this work, we successfully developed a new short and robust strategy for the synthesis of DPD which requires only one purification step. Ph-DPD was also synthesized to show the applicability of our protocol to the production of different C_1_-DPD analogs. The new strategy inspired the synthesis of 30 novel DPD-related compounds: the cycloaddition to two common precursors was employed to produce (in maximum four steps) four different small libraries where the diketo moiety of DPD was embedded in heteroaromatic rings. All the designed compounds were purified and characterized by ^1^H-NMR, ^13^C-NMR, and UHPLC-MS (purity > 90%). It is worth noting that in these compounds the open/closed equilibrium (typical of the majority of the DPD-analogs reported so far, Figure 1) is not possible. The so-obtained more stable compounds were easily purify by column chromatography. Moreover, the presence of heteroaromatic groups increases the UV absorbance and MW, rendering the compound detection by the classical analytical method (e.g., LC-MS) easier compared to previously reported analogs (e.g., ethyl-DPD).

Our new synthetic approach allowed us to synthetize a small set of racemic DPD-related compounds in a relatively easy and fast way. We demonstrated that racemic DPD is efficiently phosphorylated by LsrK, corroborating the validity of our approach. On the other hand, all compounds of our library of DPD-related did not show any activity on LsrK. Nevertheless, the synthetic procedure herein proposed might lead to the preparation of a wider compound library, thus, allowing for the discovery of a new class of LsrK inhibitors as potential antivirulence agents. Moreover, we decided to add these products to the library of MuTaLig, an innovative ligand identification platform for the drug-discovery process.

## Figures and Tables

**Table 1:** The optimization of the conditions for the oxidation of compound **3**. All the reactions were performed at room temperature.

Entry	Solvent	Oxidant and eq	Time	Yield (%)
1	Acetone	KMnO_4_/NaHCO_3_/MgSO_4_ 3.8/0.6/2.0	Overnight	No reaction
2	Acetone	KMnO_4_/NaHCO_3_/MgSO_4_ 3.9/0.6/4.2	Overnight	Traces
3	CCl_4_/ACN (1:1)	NaIO_4_/RuO_2_·H_2_O 2.2 eq/2.5% mol	3 h	Traces
4	CCl_4_/ACN (1:1)	NaIO_4_/RuO_2_·H_2_O 4.4 eq/2.5% mol	3 h	23
5	CHCl_3_/ACN/H_2_O (1:1:1)	NaIO_4_/RuO_2_·H_2_O 4.4 eq/2.5% mol	3 h	52

**Table 2:**
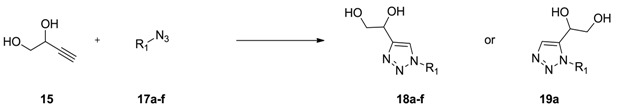
The reaction conditions to obtain **18a**–**f** and **19a**. All reactions were performed overnight at room temperature except for entry 4 where the mixture was heated at 60 °C.

Entry	R_1_	Azide, eq	Solvent	Catalyst	Product	Yield (%) ^a^	Ref.
1	(CH_2_)_2_-Ph	**17a**, 1.1	THF	CuI (10% mol) DIPEA (15% mol)	**18a**	58	[44]
2	(CH_2_)_2_-Ph	**17a**, 1.05	DCM	CuI (2% mol) DIPEA (4% mol) AcOH (cat)	**18a**	72	[48]
3	(CH_2_)_2_-Ph	**17a**, 1.0	*t*-BuOH/H_2_O (1:1)	CuSO_4_·5H_2_O (5% mol) Na Ascorbate (0.5 eq)	**18a**	89	[49]
4	(CH_2_)_2_-Ph	**17a**, 1.0	1,4-dioxane	(Cp*RuCl(PPh_3_)_2_) (2% mol)	**19a**	87	[50]
5	(CH_2_)-Ph	**17b**, 1.0	*t*-BuOH/H_2_O (1:1)	CuSO_4_·5H_2_O (5% mol) Na Ascorbate (0.5 eq)	**18b**	60	[49]
6	(CH_2_)_2_-*o*-F-Ph	**17c**, 1.0	*t*-BuOH/H_2_O (1:1)	CuSO_4_·5H_2_O (5% mol) Na Ascorbate (0.5 eq)	**18c**	62	[49]
7	(CH_2_)_2_-*m*-Pyr	**17d**, 1.0	*t*-BuOH/H_2_O (1:1)	CuSO_4_·5H_2_O (5% mol) Na Ascorbate (0.5 eq)	**18d**	88	[49]
8	(CH_2_)_5_-CN	**17e**, 1.0	*t*-BuOH/H_2_O (1:1)	CuSO_4_·5H_2_O (5% mol) Na Ascorbate (0.5 eq)	**18e**	72	[49]
9	(CH_2_)_2_-CyH	**17f**, 1.0	*t*-BuOH/H_2_O (1:1)	CuSO_4_·5H_2_O (5% mol) Na Ascorbate (0.5 eq)	**18f**	73	[49]

^a^ Isolated yield.

## Supplementary Materials

Supplementary materials are available online.
