# Supplementary material for: A Versatile Strategy for the Synthesis of 4,5-Dihydroxy-2,3-Pentanedione (DPD) and Related Compounds as Potential Modulators of Bacterial Quorum Sensing
Source: Molecules. 2018 Oct 6;23(10):2545. doi: 10.3390/molecules23102545 (PMC6222300; doi:10.3390/molecules23102545)
Supplement: Supplementary file 1 [file molecules-23-02545-s001.pdf]

Supplementary data

A versatile strategy for the synthesis of 4,5-dihydroxy-2,3-pentanedione (DPD) and related compounds as potential modulators of bacterial quorum sensing

Silvia Stotani[1,2], Viviana Gatta[3], Federico Medda[1,#], Mohan Padmanaban[1], Anna Karawajczyk[1,†], Päivi Tammela[3], Fabrizio Giordanetto[1,§], Dimitrios Tzalis[1] and Simona Collina[2,*]

[1] Medicinal Chemistry, Taros Chemicals GmbH & Co. KG, Emil-Figge-Straße 76a, 44227 Dortmund, Germany
[2] Department of Drug Sciences, Medicinal Chemistry and Pharmaceutical Technology Section, University of Pavia, Viale Taramelli 6 and 12, 27100, Pavia, Italy
[3] Centre for Drug Research, Division of Pharmaceutical Biosciences, University of Helsinki, Helsinki, Finland

#Current address: Centurion Biopharma Corporation, Engesserstraße 4, 79108 Freiburg im Breisgau, Germany

† Current address: Selvita S.A., Park Life Science, Bobrzyňskiego 14, 30-348 Krakow, Poland

§ Current address: DE Shaw Research, 120W 45th Street, New York, NY

* Correspondence: simona.collina@unipv.it; Tel.: +39 0382-987379

Table of contents

	Page
General information	S-3
Tables (chemistry)	S-4
Schemes	S-5
Synthesis of DPD: failed routes	S-6
^{1}H NMR (300 MHz, D_2O) 4,5-dihydroxy-2,3-pentanedione (DPD)	S-7
^{1}H NMR (300 MHz, D_2O) 4,5-dihydroxy-2,3-pentanedione (DPD) zoom 4.5 ppm – 1.0 ppm	S-8
^{1}H NMR (300 MHz, D_2O) 3,4-dihydroxy-1-phenylbutane-1,2-dione (Ph-DPD)	S-9
^{1}H NMR (300 MHz, D_2O) 3,4-dihydroxy-1-phenylbutane-1,2-dione (Ph-DPD) zoom 8.5 ppm – 7.0 ppm	S-10
^{1}H NMR (300 MHz, D_2O) 3,4-dihydroxy-1-phenylbutane-1,2-dione (Ph-DPD) zoom 4.7 ppm– 3.5 ppm	S-11
Characterization of compounds **17a-f**	S-12
Different conditions tested for the synthesis of triazole **18a**	S-13
Characterization of compounds **21h-k** and **22h-k**	S-14
HMBC of compound **18a**	S-15
HMBC of compound **19a**	S-16
HMBC of compound **21i**	S-17
HMBC of compound **22i**	S-18
Characterization of compounds **24l-r**	S-19
Characterization of compounds **25l-r**	S-20
Characterization of **32b**, **32s-z**	S-21
Table (biology)	S-22
Biological activity of the synthesized compounds	S-23
References	S-25

General information

Chemicals and solvents were obtained from commercial suppliers and were used without further purification. All dry reactions were performed under nitrogen atmosphere using commercial dry solvents. Flash column chromatography was performed on a silica column using 230400 mesh silica gel or Grace Reveleris X2 flash chromatography system using silica gel packed Macherey Nagel Chromabond Flash BT cartridges (60 Å, 45 μm) and Grace Reveleris flash Cartridges (60 Å, 40 μm). Thin layer chromatography was performed on Macherey Nagel precoated TLC aluminum sheets with silica gel 60 UV254 (5 μm – 17 μm). TLC visualization was accomplished by irradiation with a UV lamp (254 nm) and/or staining with $KMnO_4$ solutions. ^{1}H NMR spectra were recorded at room temperature on a Bruker Avance spectrometer operating at 300 MHz. Chemical shifts are given in ppm (δ) from tetramethylsilane as an internal standard or residual solvent peak. Significant ^{1}H NMR data are tabulated in the following order: multiplicity (s, singlet; d, doublet; t, triplet; q, quartet; m, multiplet; dd, doublet of doublets; dt, doublet of triplets; td, triplet of doublets; br, broad), coupling constant(s) in hertz, number of protons. Proton decoupled ^{13}C NMR data were acquired at 100 MHz. ^{13}C chemical shifts are reported in parts per million (δ, ppm). All NMR data were collected at room temperature (25 °C). Analytical, preparative HPLC and Electron Spray Ionization (ESI) mass spectra were performed on an Agilent UHPLC (1290 Infinity) and an Agilent Prep-HPLC (1260 Infinity) both equipped with a Diode Array Detector and a Quadrupole MS using mixture gradients of formic acid/water/acetonitrile as solvents. High-resolution electrospray ionization mass spectra (ESI-FTMS) were recorded on a Thermo LTQ Orbitrap (high-resolution mass spectrometer from Thermo Electron) coupled to an ‘Accela’ HPLC system supplied with a ‘Hypersil GOLD’ column (Termo Electron).

Tables (chemistry)

Entry	Solvent	Deprotecting agent	Temp (° C)	Time	Result
1	D_2O/DMSO-*d6* (4:1) (10 mM)	D_2SO_4 (Final 5 mM)	rt	Overnight	Decomposition
2	D_2O/DMSO-*d6* (4:1) (10 mM)	D_2SO_4 (Final 5 m)	0	Overnight	Decomposition
3	D_2O/DMSO-*d6* (4:1) (10 mM)	H_2SO_4 (cat.)	0→100	2 days	Decomposition
4	MeOD (10 mM)	D_2SO_4 (Final 5 mM)	rt	Overnight	Decomposition
5	THF	TBAF (1.1 eq)	rt	Overnight	Decomposition
6	ACN-*d3* (10 mM)	NH_4F (4.0 eq)	rt	Overnight	SM
7	ACN-*d3*/D_2O (1:1) (10 mM)	ACOD-*d3* (3.0 eq)	rt	Overnight	~10 % deprotection
8	MeOD (10 mM)	Dowex 50WX8 100-200 mesh	rt	Overnight	~30 % deprotection
9	ACN-*d3* (10 mM)	Dowex 50WX8 100-200 mesh	rt	Overnight	~30 % deprotection

Table S1: Screening of the conditions for the acidic removal of the two TBDMS groups of compound **5**.

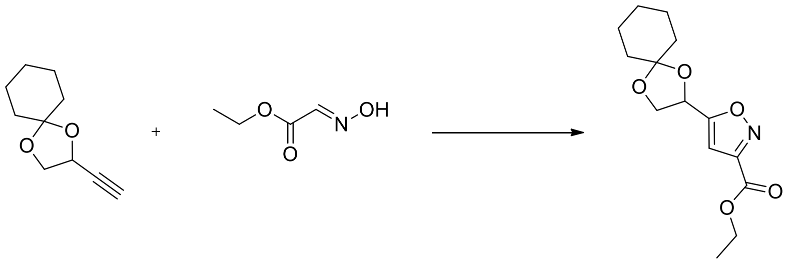

16 **27** **28**

Entry	16 (Eq)	27 (Eq)	Time (h)	Yield (%)[a]
1	1.2	1	96	16
2	1	1.2	72	18
3	1	1.5	24	21
4	1	2.0	12	36

Table S2: Different ratios of dipolarophile **16** and 1,3-dipole **27** tested for the synthesis of intermediate **28**.
[a] Isolated yield

Schemes

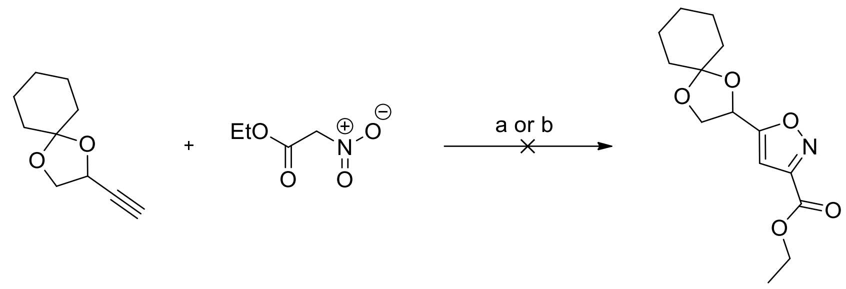

16

Ethylnitro acetate

28

Scheme S1: Attempts for the synthesis of intermediate **28**. Reagents and conditions: (a) **16** (1.0 eq), base (1.5 eq), $CHCl_3$, 60 °C, 3 days; (b) **16** (1.0 eq), PhNCO (2.0 eq), Et_3N (1.5 eq), PhMe, 100 °C, 3 days.

Synthesis of DPD: failed routes

Synthesis of 3 and 4: To a stirred suspension of NaH (2.0 eq) in THF was added **2** (1.0 eq). The suspension was stirred at room temperature for 45 min and afterwards it was cooled to 0 °C using an ice bath. TBDMSCl (or TMSCl) (1.3 eq) in THF was added dropwise. The reaction was vigorously stirred at room temperature for 2 hours. The mixture was poured **slowly** onto a cold solution of aqueous K_2CO_3 (10%) and extracted three times with Et_2O. The organic layer was dried over $MgSO_4$, filtered and concentrated *in vacuo* to yield **3** as a yellowish oil (90%) or 4 as a colorless oil (92%).

2,2,3,3,8,8,9,9-octamethyl-5-(prop-1-yn-1-yl)-4,7-dioxa-3,8-disiladecane (3): yellowish oil, 90%, R_f = 0.65 (CyH/EtOAc 9:1). ^{1}H NMR (300 MHz, $CDCl_3$) δ 4.37 – 4.33 (m, 1H), 3.63 (s, 1H), 3.61 (s, 1H), 1.81 (d, *J* = 2.1 Hz, 3H), 0.90 (d, *J* = 4.3 Hz, 18H), 0.11 (d, *J* = 5.9 Hz, 6H), 0.07 (d, *J* = 2.2 Hz, 6H) ppm.

2,2,7,7,8,8-hexamethyl-4-(prop-1-yn-1-yl)-3,6-dioxa-2,7-disilanonane (4): colorless oil, 92%, R_f = 0.73 (CyH/EtOAc 9:1). ^{1}H NMR (300 MHz, $CDCl_3$) δ 4.39 – 4.31 (m, 1H), 3.64 – 3.62 (m, 1H), 3.60 – 3.56 (m, 1H), 1.85 – 1.81 (m, 3H), 0.91 (d, *J* = 3.3 Hz, 9H), 0.17 (s, 6H), 0.12 – 0.07 (m, 9H) ppm.

Synthesis of 5 and 6: To a stirred solution of **3** (or **4**) (1.0 eq) in a 1:1:1 mixture of $CHCl_3$/ACN/H_2O was added $NaIO_4$ (4.4 eq) and RuO_2*H_2O (2.5% mol). The mixture was vigorously stirred for 3 hours. Solvent was evaporated under reduced pressure, the residue was redissolved in EtOAc and filtered through a silica pad. The eluate was extracted three times with water, dried over $MgSO_4$, filtered and concentrated *in vacuo*. Flash chromatography using CyH/EtOAc (3:1) afforded **5** as a yellow oil (52%) and **6** as a yellow oil (65%).

4,5-bis[(*t*-butyldimethylsilyl)oxy]pentane-2,3-dione (5): yellow oil, 52%, R_f = 0.25 (CyH/EtOAc 9:1). ^{1}H NMR (300 MHz, $CDCl_3$) δ 4.96 (t, *J* = 4.9 Hz, 1H), 4.00 (dd, *J* = 5.0 Hz, *J* = 10.3 Hz, 1H), 3.76 (dd, *J* = 4.7 Hz, *J* = 10.3 Hz, 1H), 2.32 (s, 3H), 0.89 (s, 10H), 0.85 (s, 8H), 0.09 – 0.07 (m, 4H), 0.04 – 0.03 (m, 8H) ppm.

5-[(*t*-butyldimethylsilyl)oxy]-4-[(trimethylsilyl)oxy]pentane-2,3-dione (6): yellow oil, 65%, R_f = 0.75 ($CHCl_3$/MeOH 5:1). ^{1}H NMR (300 MHz, $CDCl_3$) δ 4.90 (s, 1H), 4.14 (dd, *J* = 3.0 Hz, *J* = 10.8 Hz, 1H), 3.86 (dd, *J* = 2.8 Hz, *J* = 10.8 Hz, 1H), 2.39 (s, 3H), 0.91 (s, 9H), 0.83 (s, 9H), 0.01 (d, *J* = 8.3 Hz, 6H) ppm.

^{1}H NMR (300 MHz, D_2O) 4,5-dihydroxy-2,3-pentanedione (DPD)

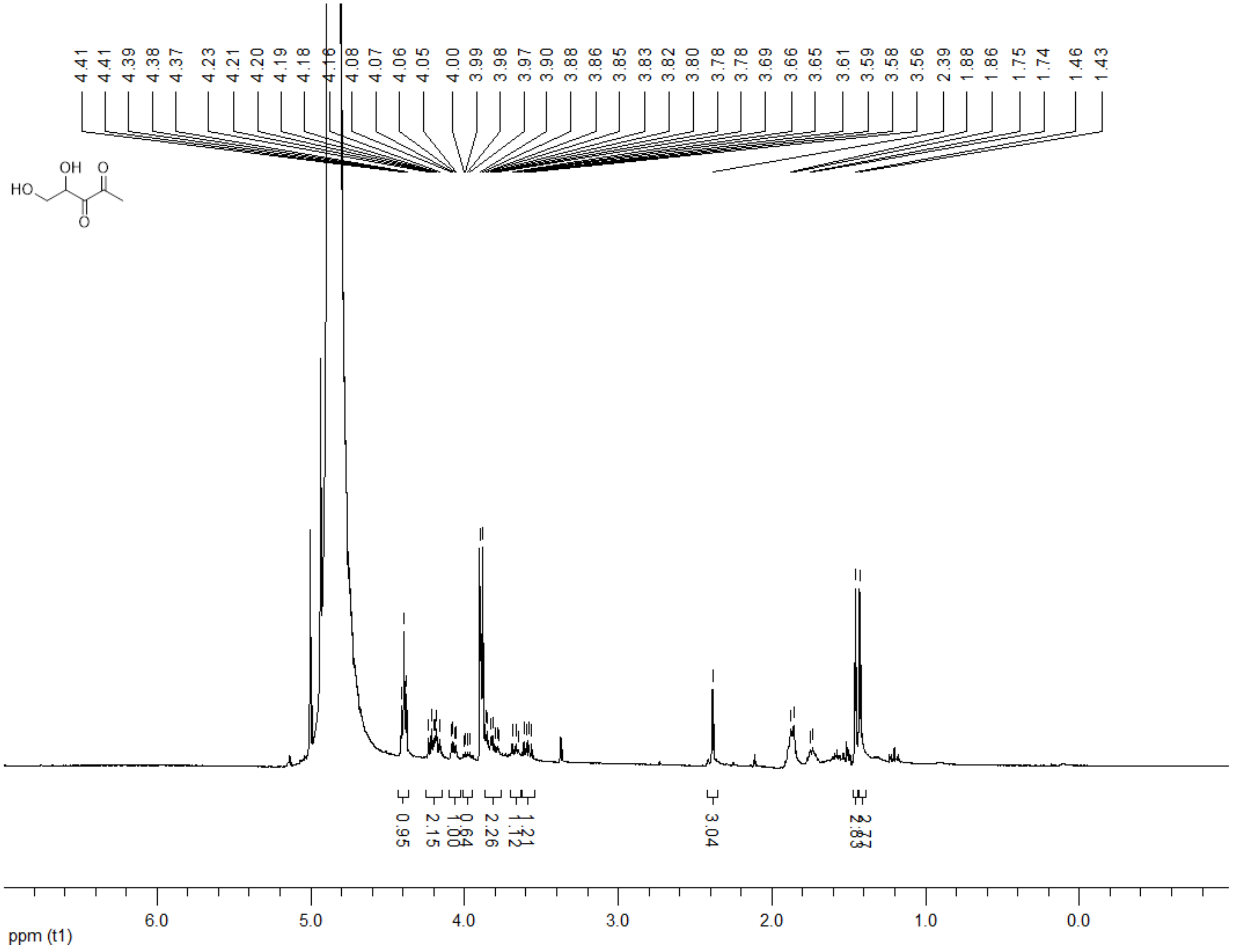

^{1}H NMR (300 MHz, D_2O) 4,5-dihydroxy-2,3-pentanedione (DPD) zoom 4.5 ppm – 1.0 ppm

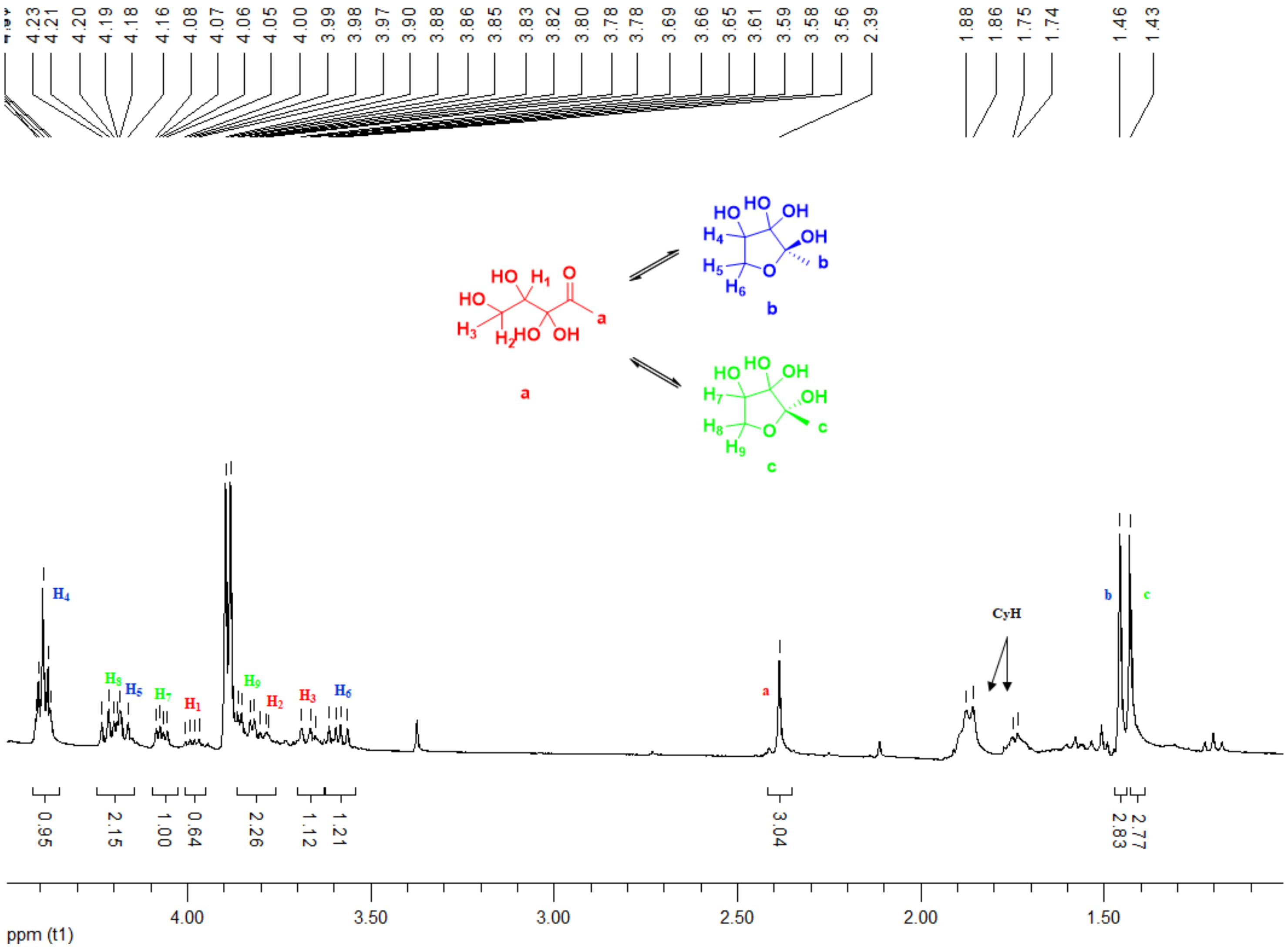

^{1}H NMR (300 MHz, D_2O) 3,4-dihydroxy-1-phenylbutane-1,2-dione (Ph-DPD)

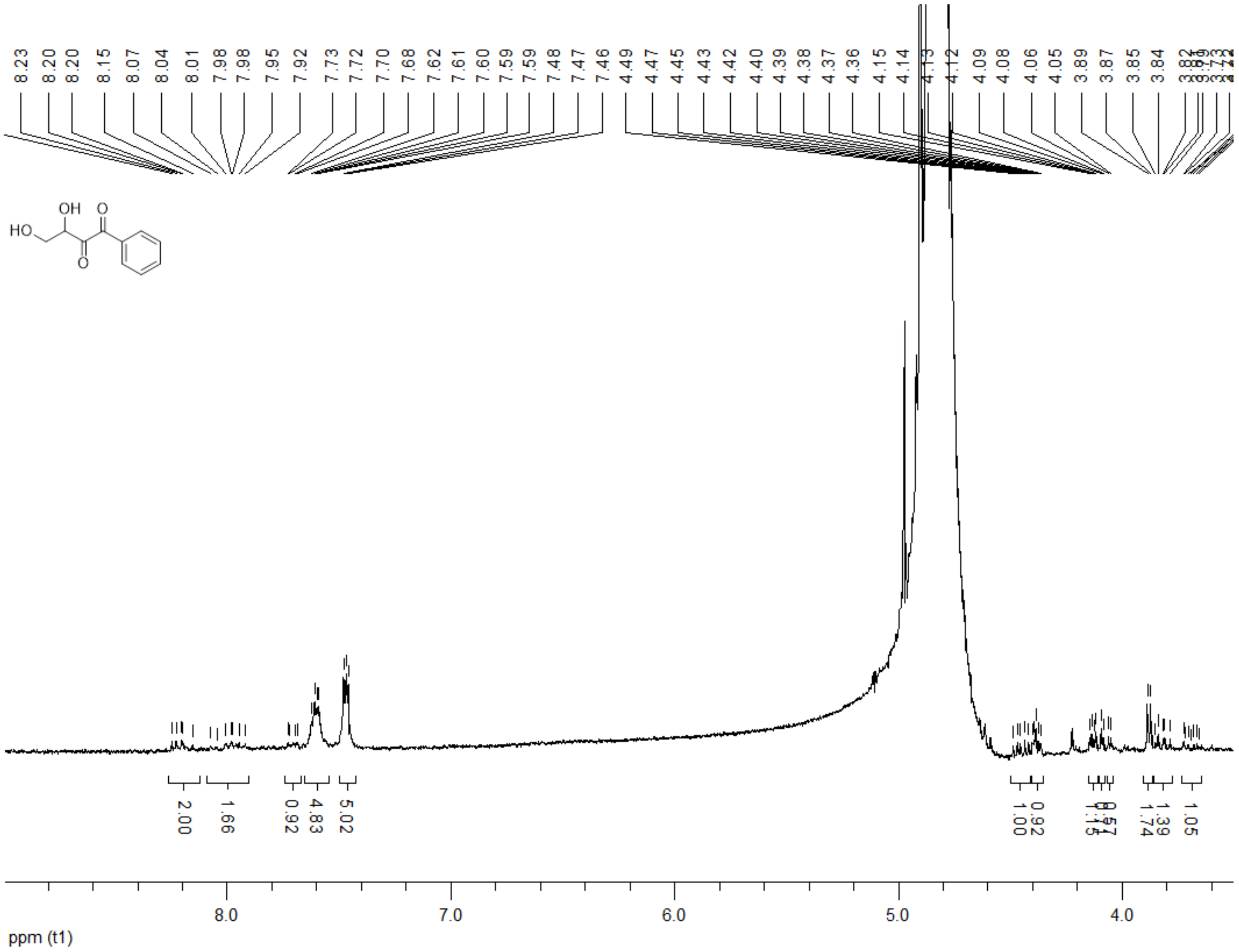

¹H NMR (300 MHz, D_2O) 3,4-dihydroxy-1-phenylbutane-1,2-dione (Ph-DPD) zoom 8.5 ppm – 7.0 ppm

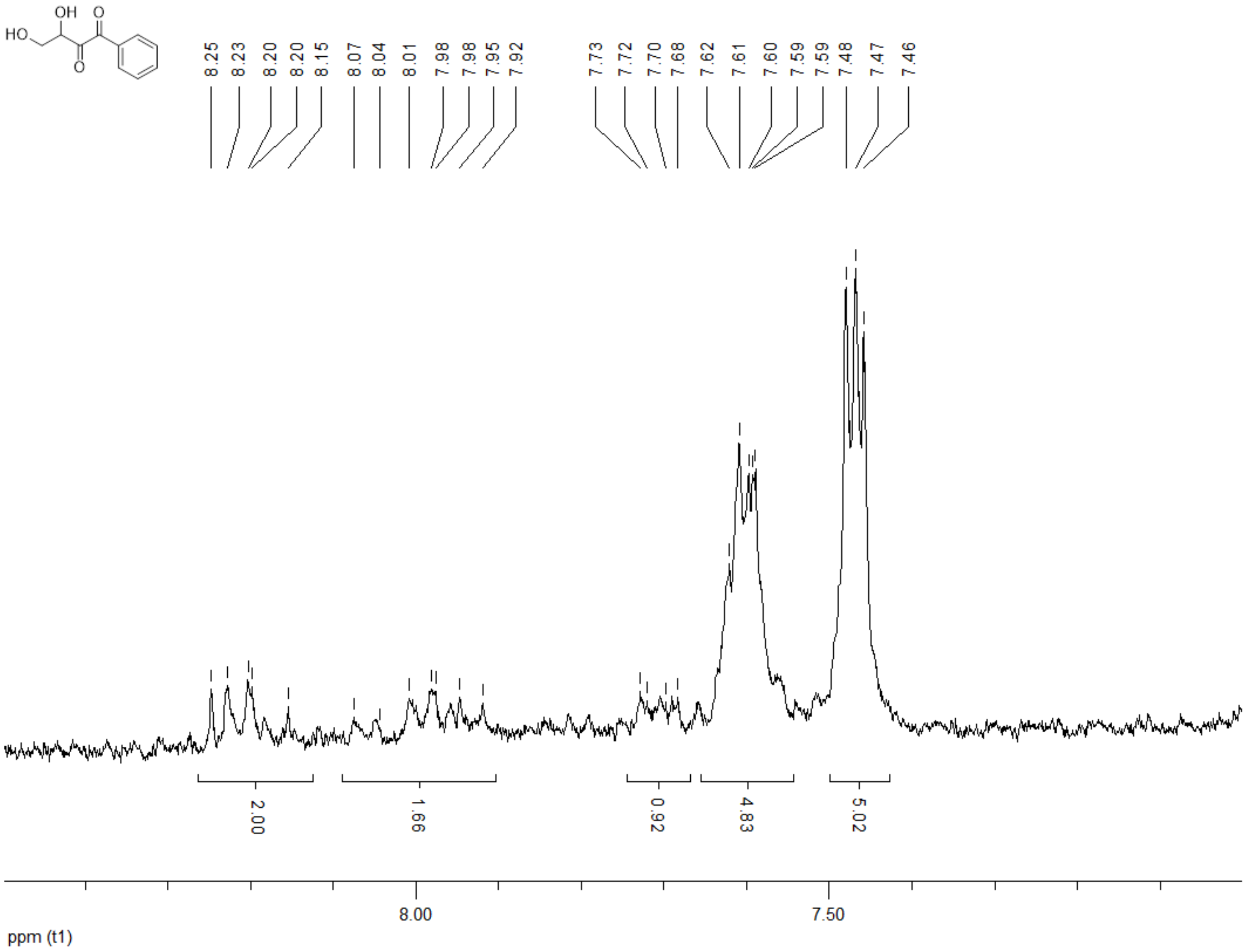

^{1}H NMR (300 MHz, D_2O) 3,4-dihydroxy-1-phenylbutane-1,2-dione (Ph-DPD) zoom 4.7 ppm– 3.5 ppm

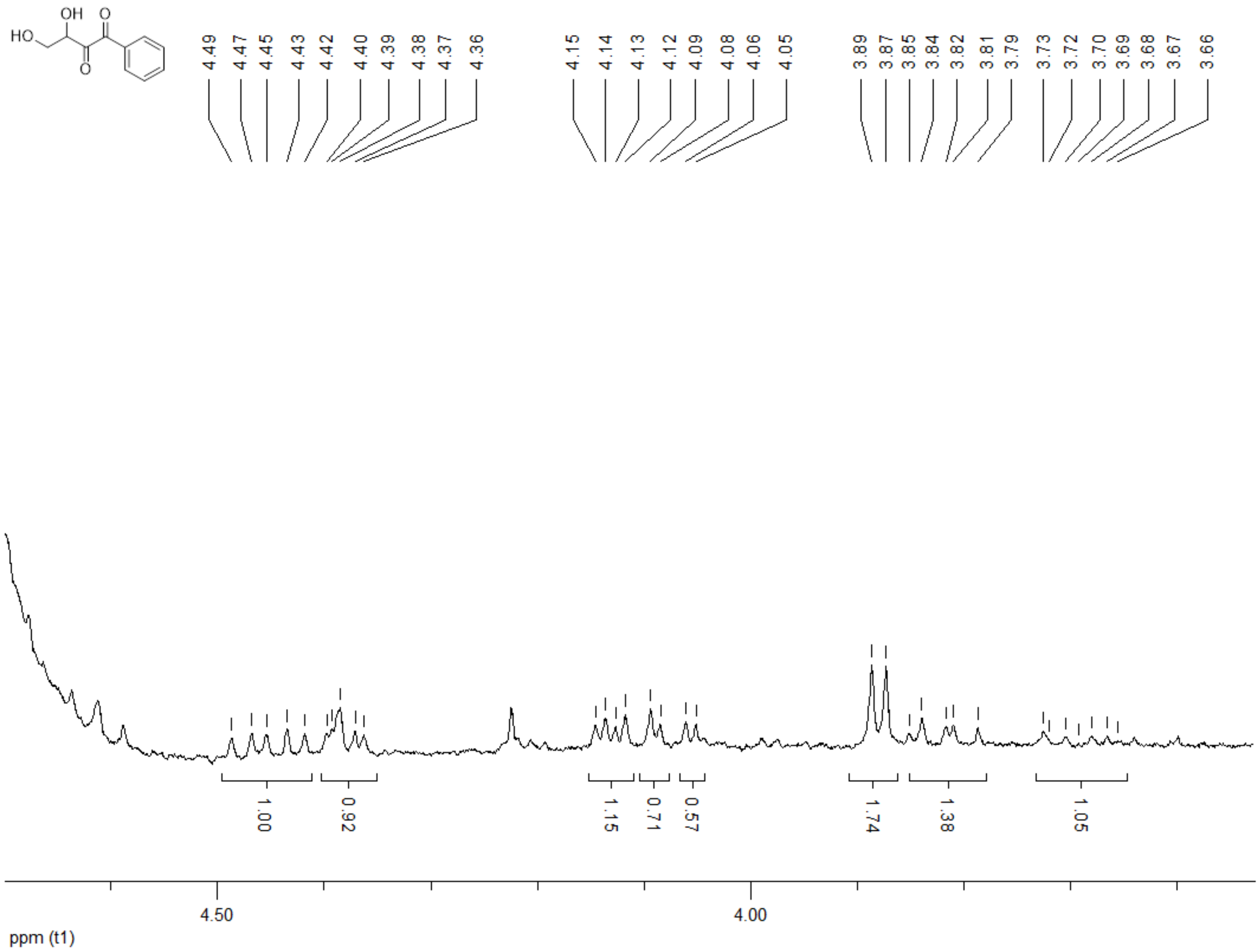

Characterization of compounds 17a-f

(2-azidoethyl)benzene (17a): yellow oil, 85%. ^{1}H NMR (300 MHz, $CDCl_3$) δ 7.31 – 7.28 (m, 2H), 7.23 – 7.17 (m, 3H), 3.46 (dt, *J* = 2.8 Hz, *J* = 7.2 Hz, 2H), 2.85 (t, *J* = 7.3 Hz, 2H) ppm; ^{13}C NMR (100 MHz, $CDCl_3$) δ 138.0, 128.7, 128.6, 126.7, 52.4, 35.3 ppm [1].

(azidomethyl)benzene (17b): colorless oil, 70%. ^{1}H NMR (300 MHz, $CDCl_3$) δ 7.44 – 7.32 (m, 5H), 4.35 (s, 2H) ppm; ^{13}C NMR (100 MHz, $CDCl_3$) δ 135.4, 128.8, 128.3, 128.2, 54.8 ppm [2].

1-(2-azidoethyl)-2-fluorobenzene (17c) colorless oil, 86%. ^{1}H NMR (300 MHz, $CDCl_3$) δ 7.26 (t, *J* = 7.2 Hz, 2H), 7.15 – 7.04 (m, 2H), 3.54 (t, *J* = 7.1 Hz, 2H), 2.97 (t, *J* = 7.2 Hz, 2H) ppm;^{13}C NMR (100 MHz, $CDCl_3$) δ 161.2 (d, *J* = 245.5 Hz), 131.1 (d, *J* = 4.8 Hz), 128.7 (d, *J* = 8.2 Hz), 124.9 (d, *J* = 15.8 Hz), 124.2 (d, *J* = 3.5 Hz), 115.5 (d, *J* = 22.0 Hz), 51.1 (d, *J* = 1.5 Hz), 29.0 (d, *J* = 2.2 Hz) ppm [3]

2-(2-azidoethyl)pyridine (17d): yellowish oil, 30%. ^{1}H NMR (300 MHz, $CDCl_3$) δ 8.55 (d, *J* = 4.4 Hz, 1H), 7.62 (dt, *J* = 1.8 Hz, *J* = 7.7 Hz, 1H), 7.21 – 7.14 (m, 2H), 3.71 (t, *J* = 6.9 Hz, 2H), 3.05 (t, *J* = 6.9 Hz, 2H) ppm; ^{13}C NMR (100 MHz, $CDCl_3$) δ 158.0, 149.5, 136.5, 123.5, 121.8, 50.6, 37.5 ppm [4].

6-azidohexanenitrile (17e): colorless oil, 48%. ^{1}H NMR (300 MHz, $CDCl_3$) δ 3.30 (t, *J* = 6.5 Hz, 2H), 2.36 (t, *J* = 6.9 Hz, 2H), 1.74 – 1.50 (m, 6H) ppm; ^{13}C NMR (100 MHz, $CDCl_3$) δ 119.4, 51.0, 28.1, 25.8, 25.0, 17.1 ppm [5].

(2-azidoethyl)cyclohexane (17f): colorless oil, 64%. ^{1}H NMR (300 MHz, $CDCl_3$) δ 3.28 (t, *J* = 7.2 Hz, 2H), 1.72 – 1.64 (m, 5H), 1.49 (dd, *J* = 7.0 Hz, *J* = 14.1 Hz, 2H), 1.40 – 1.30 (m, 1H), 1.26 – 1.12 (m, 3H), 0.91 (q, *J* = 11.5 Hz, 2H) ppm; ^{13}C NMR (100 MHz, $CDCl_3$) δ 49.2, 36.0, 35.0, 33.0, 26.4, 26.1 ppm [6].

Different conditions tested for the synthesis of triazole 18a

A) To a stirred solution of **15** (1.0 eq) in THF was added CuI (10% mol), DIPEA (15% mol) and (2-azidoethyl)benzene (**17a**) (1.1 eq). The mixture was stirred at room temperature overnight. Solvent was evaporated under reduced pressure, the crude was redissolved in ACN (1 mL), filtered and purified by preparative HPLC to yield **18a** as an orange oil (58%) [7].

B) To a stirred solution of **15** (1.0 eq), CuI (2%mol), DIPEA (15%mol) and AcOH (cat.) in DCM was added (2-azidoethyl)benzene (**17a**) (1.05 eq). The mixture was stirred at room temperature overnight. Solvent was evaporated under reduced pressure, the crude was redissolved in ACN (1 mL), filtered and purified by preparative HPLC to yield **18a** as an orange oil (72%) [8].

C) To a stirred solution of **15** (1.0 eq) in a 1:1 mixture of H_2O/*t*-BuOH were added (2-azidoethyl)benzene (**17a**) (1.0 eq), sodium ascorbate (0.5 eq) and $CuSO_4$*5H_2O (5% mol). The reaction was stirred at room temperature overnight. Solvent was evaporated under reduced pressure, the crude was redissolved in ACN (1 mL), filtered and purified by preparative HPLC to yield **18a** as an orange oil (89%) [9].

Characterization of compounds 21h-k and 22h-k

4-{1,4-dioxaspiro[4.5]decan-2-yl}-1-methyl-1*H*-1,2,3-triazole (21h): yellowish oil, 53%, R_f = 0.48 (CyH/EtOAC 3:1), UHPLC-ESI-MS: R_t = 2.30, *m/z* = 224.2 [M + H]$^+$. ^{1}H NMR (300 MHz, $CDCl_3$) δ 7.59 (s, 1H), 5.20 (t, *J* = 6.4 Hz, 1H), 4.35 (dd, *J* = 6.4 Hz, *J* = 8.5 Hz, 1H), 4.11 (s, 3H), 4.06 (dd, *J* = 6.5 Hz, *J* = 8.5 Hz, 1H), – 1.56 (m, 8H), 1.42 – 1.41 (m, 2H) ppm; ^{13}C NMR (100 MHz, $CDCl_3$) δ 135.3, 131.9, 111.5, 67.9, 67.5, 35.9, 35.4, 34.9, 24.9, 23.9, 23.8 ppm.

5-{1,4-dioxaspiro[4.5]decan-2-yl}-1-methyl-1*H*-1,2,3-triazole (22h): yellowish oil, 62%, R_f = 0.50 (CyH/EtOAC 3:1), UHPLC-ESI-MS: R_t = 2.54, *m/z* = 224.0 [M + H]$^+$. ^{1}H NMR (300 MHz, $CDCl_3$) δ 7.57 (s, 1H), 5.22 (t, *J* = 6.6 Hz, 1H), 4.32 (dd, *J* = 6.3 Hz, *J* = 8.3 Hz, 1H), 4.16 (s, 3H), 3.99 (dd, *J* = 7.0 Hz, *J* = 8.3 Hz, 1H), – 1.60 (m, 8H), 1.44 – 1.41 (m, 2H) ppm; ^{13}C NMR (100 MHz, $CDCl_3$) δ 147.5, 132.4, 110.7, 70.2, 69.3, 41.6, 36.0, 35.2, 25.1, 23.9, 23.8 ppm.

1-(cyclopropylmethyl)-4-{1,4-dioxaspiro[4.5]decan-2-yl}-1*H*-1,2,3-triazole (21i): colorless oil, 47%, R_f = 0.29 ($CHCl_3$/MeOH 9:1), UHPLC-ESI-MS: R_t = 2.61, *m/z* = 264.2 [M + H]$^+$. ^{1}H NMR (300 MHz, $CDCl_3$) δ 7.63 (s, 1H), 5.29 (t, *J* = 6.6 Hz, 1H), 4.36 (t, *J* = 7.3 Hz, 1H), 4.18 (d, *J* = 7.2 Hz, 2H), 4.03 (t, *J* = 7.6 Hz, 1H), 1.69 – 1.62 (m, 8H), 1.41 – 1.39 (m, 2H), 1.28 – 1.25 (m, 1H), 0.68 (d, *J* = 7.3 Hz, 2H), 0.42 (d, *J* = 4.3 Hz, 2H) ppm; ^{13}C NMR (100 MHz, $CDCl_3$) δ 147.5, 120.9, 110.4, 70.6, 69.4, 54.9, 36.1, 35.1, 25.0, 23.9, 23.8, 10.9, 4.2 (d, *J* = 4.1 Hz) ppm.

1-(cyclopropylmethyl)-5-{1,4-dioxaspiro[4.5]decan-2-yl}-1*H*-1,2,3-triazole (22i): colorless oil, 42%, R_f = 0.31 ($CHCl_3$/MeOH 9:1), UHPLC-ESI-MS: R_t = 2.72, *m/z* = 264.2 [M + H]$^+$. ^{1}H NMR (300 MHz, $CDCl_3$) δ 7.59 (s, 1H), 5.21 (t, *J* = 6.4 Hz, 1H), 4.36 (dd, *J* = 6.3 Hz, *J* = 8.4 Hz, 1H), 4.28 (d, *J* = 7.2 Hz, 2H), 4.07 (dd, *J* = 6.6 Hz, *J* = 8.4 Hz, 1H), 1.65 – 1.60 (m, 8H), 1.42 – 1.38 (m, 3H), 0.63 (d, *J* = 8.1 Hz, 2H), 0.48 (d, *J* = 4.8 Hz, 2H) ppm; ^{13}C NMR (100 MHz, $CDCl_3$) δ 134.8, 131.6, 111.4, 68.2, 67.4, 53.3, 35.9, 35.0, 24.9, 23.8, 11.3, 4.5, 4.0 ppm.

1-butyl-4-{1,4-dioxaspiro[4.5]decan-2-yl}-1*H*-1,2,3-triazole (21j): colorless oil, 37%, R_f = 0.23 (CyH/EtOAC 3:1), UHPLC-ESI-MS: R_t = 2.77, *m/z* = 266.2 [M + H]$^+$. ^{1}H NMR (300 MHz, $CDCl_3$) δ 7.51 (s, 1H), 5.29 (t, *J* = 6.6 Hz, 1H), 4.39 – 4.31 (m, 3H), 4.02 (dd, *J* = 7.0 Hz, *J* = 8.2 Hz, 1H), 1.88 (td, *J* = 7.4 Hz, *J* = 14.9 Hz, 2H), 1.70 – 1.62 (m, 8H), 1.44 – 1.32 (m, 4H), 0.95 (t, *J* = 7.3 Hz, 3H) ppm; ^{13}C NMR (100 MHz, $CDCl_3$) δ 147.7, 121.2, 110.4, 70.7, 69.5, 50.1, 36.2, 35.2, 32.2, 25.1, 24.0, 23.8, 19.7, 13.4 ppm.

1-butyl-4-{1,4-dioxaspiro[4.5]decan-2-yl}-1*H*-1,2,3-triazole (22j): colorless oil, 31%, R_f = 0.26 (CyH/EtOAC 3:1), UHPLC-ESI-MS: R_t = 2.89, *m/z* = 266.2 [M + H]$^+$. ^{1}H NMR (300 MHz, $CDCl_3$) δ 7.59 (s, 1H), 5.17 (t, *J* = 6.5 Hz, 1H), 4.43 – 4.33 (m, 3H), 4.06 (dd, *J* = 6.6 Hz, *J* = 8.4 Hz, 1H), 1.93 (td, *J* = 7.4 Hz, *J* = 15.1 Hz, 2H), 1.69 – 1.58 (m, 8H), 1.43 – 1.36 (m, 4H), 0.97 (t, *J* = 7.3 Hz, 3H) ppm; ^{13}C NMR (100 MHz, $CDCl_3$) δ 144.8, 135.1, 111.5, 68.2, 67.5, 48.6, 36.0, 35.1, 32.2, 25.0, 23.9, 19.8, 13.6 ppm.

4-{1,4-dioxaspiro[4.5]decan-2-yl}-1-(2-ethoxyethyl)-1*H*-1,2,3-triazole (21k): colorless oil, 38%, R_f = 0.21 (CyH/EtOAC 3:1), UHPLC-ESI-MS: R_t = 2.51, *m/z* = 282.2 [M + H]$^+$. ^{1}H NMR (300 MHz, $CDCl_3$) δ 7.69 (s, 1H), 5.30 (t, *J* = 6.6 Hz, 1H), 4.51 (t, *J* = 5.1 Hz, 2H), 4.37 (dd, *J* = 6.3 Hz, *J* = 8.3 Hz, 1H), 4.06 (dd, *J* = 6.9 Hz, *J* = 8.3 Hz, 1H), 3.78 (t, *J* = 5.4 Hz, 2H), 3.48 (t, *J* = 7.0 Hz, 2H), 1.72 – 1.62 (m, 8H), 1.45 – 1.41 (m, 2H), 1.17 (t, *J* = 7.0 Hz, 3H) ppm; ^{13}C NMR (100 MHz, $CDCl_3$) δ 147.6, 122.7, 110.5, 70.7, 69.5, 68.7, 66.8, 50.5, 36.2, 35.3, 25.1, 24.0, 23.9, 15.0 ppm.

4-{1,4-dioxaspiro[4.5]decan-2-yl}-1-(2-ethoxyethyl)-1*H*-1,2,3-triazole (22k): colorless oil, 29%, R_f = 0.23 (CyH/EtOAC 3:1), UHPLC-ESI-MS: R_t = 2.64, *m/z* = 282.2 [M + H]$^+$. ^{1}H NMR (300 MHz, $CDCl_3$) δ 7.61 (s, 1H), 5.31 (t, *J* = 6.3 Hz, 1H), 4.59 – 4.55 (m, 2H), 4.35 (dd, *J* = 6.3 Hz, *J* = 8.4 Hz, 1H), 4.03 (dd, *J* = 6.5 Hz, *J* = 8.4 Hz, 1H), 3.85 – 3.80 (m, 2H), 3.46 – 3.41 (m, 2H), 1.66 – 1.57 (m, 8H), 1.43 – 1.41 (m, 2H), 1.12 (t, *J* = 7.0 Hz, 3H) ppm; ^{13}C NMR (100 MHz, $CDCl_3$) δ 146.0, 122.1, 111.2, 69.2, 68.7, 67.8, 66.8, 48.7, 36.0, 35.1, 25.0, 23.9, 15.0 ppm..

HMBC of compound 18a

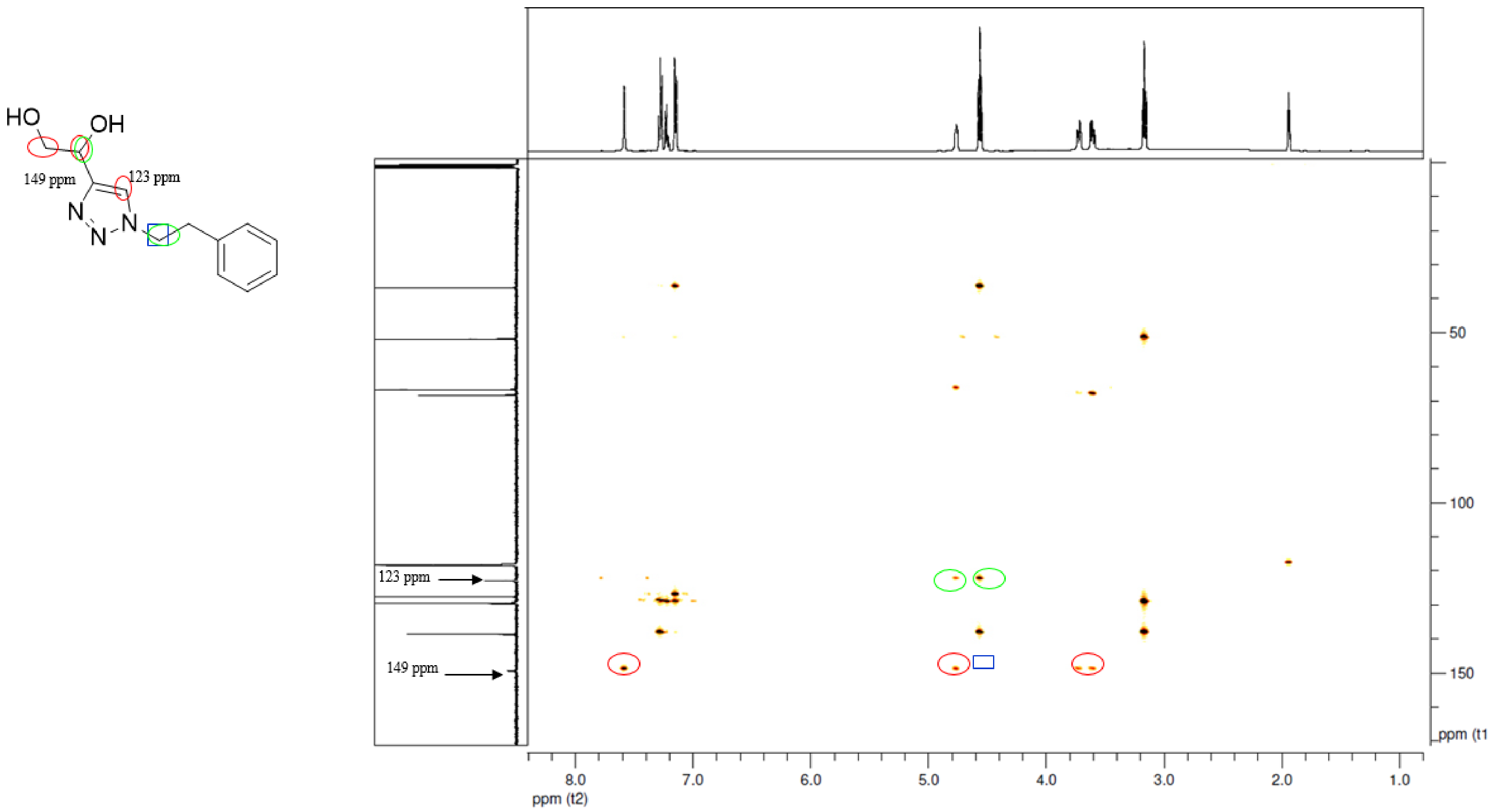

HMBC of compound 19a

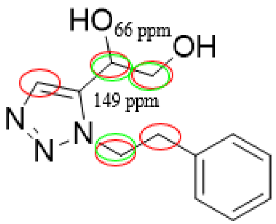

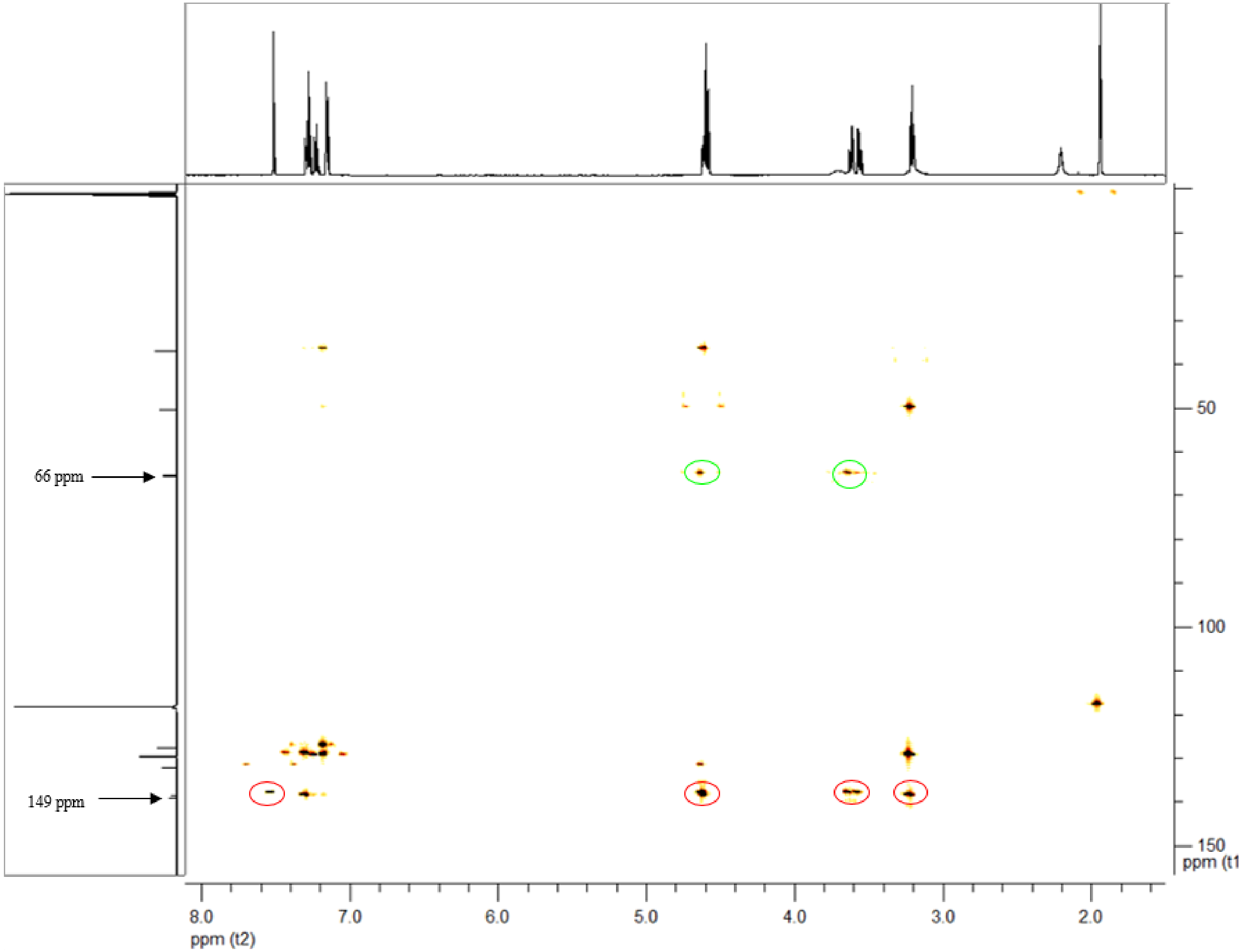

HMBC of compound 21i

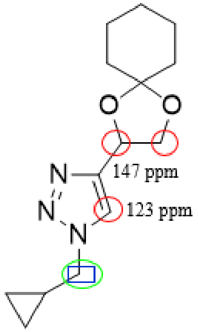

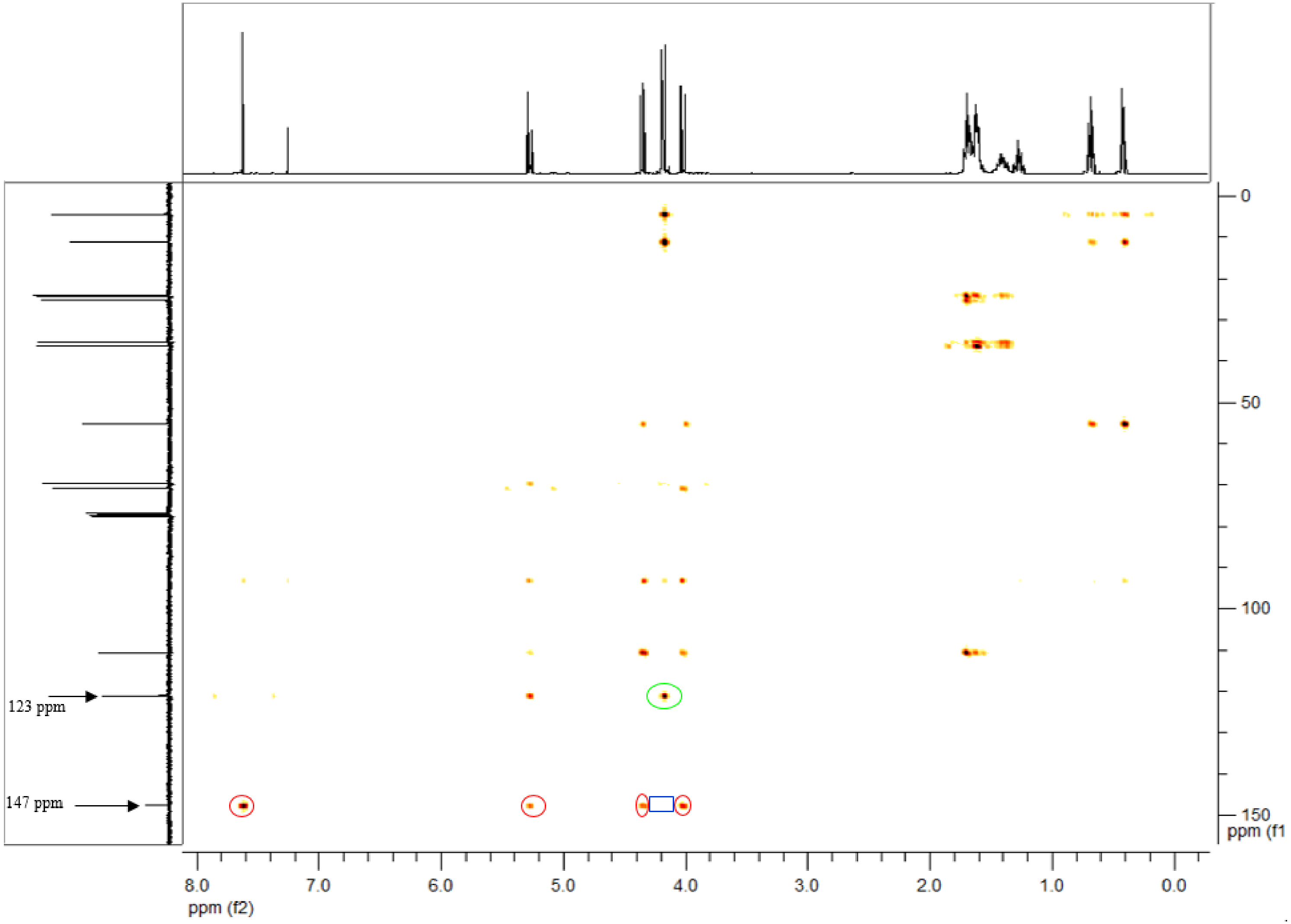

HMBC of compound 22i

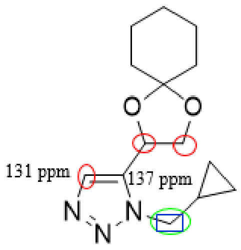

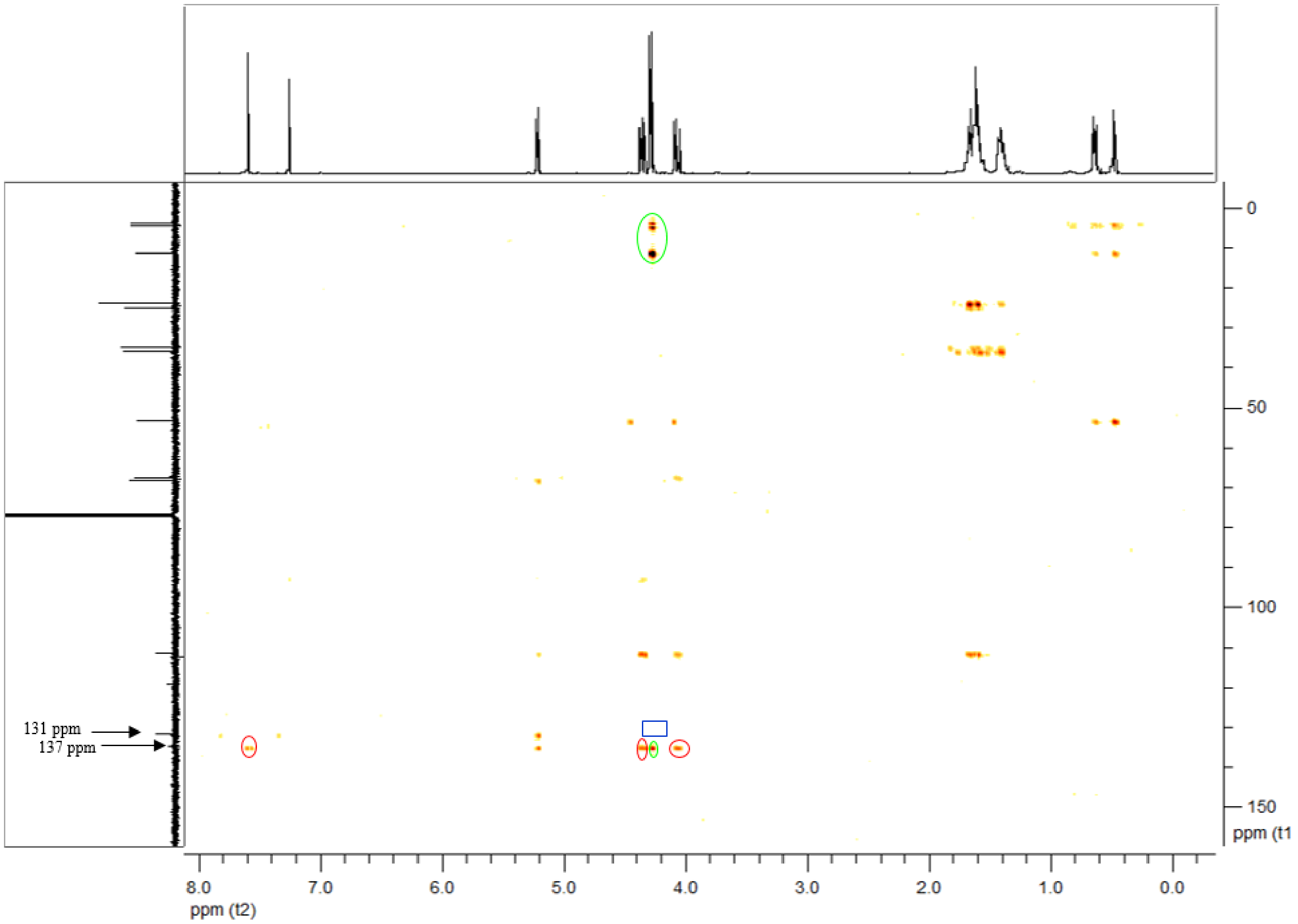

Characterization of compounds 24l-r

(*E*)-*N*-[(4-methylphenyl)methylidene]hydroxylamine (24l): brownish solid, 90%, R_f = 0.68 ($CHCl_3$/MeOH 9:1). ^{1}H NMR (300 MHz, $CDCl_3$) δ 8.67 (s br, 1H), 8.14 (s, 1H), 7.48 (d, *J* = 8.1 Hz, 2H), 7.20 (d, *J* = 7.9 Hz, 2H), 2.38 (s, 3H) ppm; ^{13}C NMR (100 MHz, $CDCl_3$) δ 150.3, 140.3, 129.5, 129.2, 127.0, 21.4 ppm [10].

(*E*)-*N*-[(3-chlorophenyl)methylidene]hydroxylamine (24m): white solid, 98%, R_f = 0.70 ($CHCl_3$/MeOH 9:1). ^{1}H NMR (300 MHz, $CDCl_3$) δ 8.10 (s, 1H), 7.59 (d, *J* = 1.8 Hz, 1H), 7.44 (td, *J* = 1.6 Hz, *J* = 7.0 Hz, 1H), 7.39 – 7.29 (m, 2H) ppm; ^{13}C NMR (100 MHz, $CDCl_3$) δ 149.1, 134.9, 133.8, 130.0, 126.8, 125.2 ppm [10].

(*E*)-*N*-[(2,4-difluorophenyl)methylidene]hydroxylamine (24n): white solid, 90%, R_f = 0.74 ($CHCl_3$/MeOH 9:1). ^{1}H NMR (300 MHz, $CDCl_3$) δ 8.31 (s, 1H), 7.73 (dt, *J* = 6.5 Hz, *J* = 8.4 Hz, 1H), 6.94 – 6.82 (m, 2H) ppm; ^{13}C NMR (100 MHz, $CDCl_3$) δ 164.2 (dd, *J* = 12.1 Hz, *J* = 217.2 Hz), 160.8 (dd, *J* = 12.0 Hz, *J* = 219.3 Hz), 143.6, 128.3 (dd, *J* = 4.4 Hz, *J* = 9.8 Hz), 116.3 (dd, *J* = 4.0 Hz, *J* = 11.0 Hz), 112.2 (dd, *J* = 3.6 Hz, *J* = 21.9 Hz), 104.4 (t, *J* = 25.3 Hz) ppm [11].

(*E*)-*N*-[(pyridin-3-yl)methylidene]hydroxylamine (24o): white solid, 83%, R_f = 0.47 ($CHCl_3$/MeOH 9:1). ^{1}H NMR (300 MHz, DMSO-d_6) δ 11.56 (s, 1H), 8.75 (d, *J* = 1.8 Hz, 1H), 8.55 (dd, *J* = 1.6 Hz, *J* = 4.8 Hz, 1H), 8.20 (s, 1H), 7.99 (td, *J* = 1.8 Hz, *J* = 7.9 Hz, 1H), 7.42 (dd, *J* = 4.8 Hz, *J* = 7.9 Hz, 1H) ppm; ^{13}C NMR (100 MHz, DMSO-d_6) δ 149.9, 147.7, 145.6, 133.0, 128.9, 123.8 ppm [12].

(*E*)-*N*-(cyclopropylmethylidene)hydroxylamine (24p): white solid, 77%, R_f = 0.66 ($CHCl_3$/MeOH 9:1). The compound was obtained as a mixture of *syn* and *anti* oximes. The mixture was not separated and the compound was used for further reaction without purification. Only the **major** isomer is reported. ^{1}H NMR (300 MHz, $CDCl_3$) δ 6.02 (d, *J* = 8.8 Hz, 1H), 2.34 – 2.23 (m, 1H), 0.98 – 0.91 (m, 2H), 0.65 – 0.60 (m, 2H) ppm; ^{13}C NMR (100 MHz, $CDCl_3$) δ 155.4, 10.8, 6.0, 5.5 ppm [13].

(*E*)-*N*-[(oxolan-3-yl)methylidene]hydroxylamine (24q): yellowish oil, 76%, R_f = 0.64 ($CHCl_3$/MeOH 9:1). The compound was obtained as a mixture of *syn* and *anti* oximes. The mixture was not separated and the compound was used for further reaction without purification. Only the **major** isomer is reported (not all the peaks are integrated and peaked in order to make the NMR picture easier to understand). ^{1}H NMR (300 MHz, $CDCl_3$) δ 8.68 (s br), 7.38 (d, *J* = 7.2 Hz, 1H), 3.96 – 3.93 (m, 1H), 3.84 – 3.75 (m, 2H), 3.70 – 3.67 (m, 1H), 3.11 – 2.99 (m, 1H), 2.17 – 2.10 (m, 1H), 1.95 – 1.87 (m, 1H) ppm; ^{13}C NMR (100 MHz, $CDCl_3$) δ 152.1, 70.6, 68.0, 39.5, 30.5 ppm [14].

(*E*)-*N*-(cyclohexylmethylidene)hydroxylamine (24r): yellowish oil, 98%, R_f = 0.58 ($CHCl_3$/MeOH 9:1). The compound was obtained as a mixture of *syn* and *anti* oximes. The mixture was not separated and the compound was used for further reaction without purification. Only the **major** isomer is reported. ^{1}H NMR (300 MHz, $CDCl_3$) δ 7.32 (d, *J* = 6.1 Hz, 1H), 2.25 – 2.16 (m, 1H), 1.81 – 1.15 (m, 15 H, major and minor) ppm; ^{13}C NMR (100 MHz, $CDCl_3$) δ 155.9, 38.4, 30.1, 25.8, 25.4 ppm [15].

Characterization of compounds 25l-r

(*Z*)-*N*-hydroxy-4-methylbenzene-1-carbonimidoyl chloride (25l): whitish solid, 75%, R_f = 0.64 (CyH/EtOAc 3:1). ^{1}H NMR (300 MHz, $CDCl_3$) δ 8.74 (s br, 1H), 7.73 (d, *J* = 8.3 Hz, 2H), 7.21 (d, *J* = 8.0 Hz, 2H), 2.39 (s, 3H) ppm; ^{13}C NMR (100 MHz, $CDCl_3$) δ 150.7, 141.1, 140.4, 129.2, 127.1, 21.3 ppm [16].

(*Z*)-3-chloro-*N*-hydroxybenzene-1-carbonimidoyl chloride (25m): white solid, 80%, R_f = 0.66 (CyH/EtOAc 3:1). ^{1}H NMR (300 MHz, $CDCl_3$) δ 8.61 (s, 1H), 7.83 (t, *J* = 1.7 Hz, 1H), 7.72 (ddd, *J* = 1.3 Hz, *J* = 1.8 Hz, *J* = 7.8 Hz, 1H), 7.42 (ddd, *J* = 1.2 Hz, *J* = 1.9 Hz, *J* = 8.0 Hz, 1H), 7.34 (t, *J* = 7.9 Hz, 1H) ppm; ^{13}C NMR (100 MHz, $CDCl_3$) δ 138.8, 134.6, 134.1, 130.7, 129.7, 127.2, 125.3 ppm [17].

(*Z*)-2,4-difluoro-*N*-hydroxybenzene-1-carbonimidoyl chloride (25n): white solid, 73%, R_f = 0.66 (CyH/EtOAc 3:1). ^{1}H NMR (300 MHz, $CDCl_3$) δ 8.81 (s, 1H), 7.67 (dt, *J* = 6.3 Hz, *J* = 8.4 Hz 1H), 7.00 – 6.88 (m, 2H) ppm; ^{13}C NMR (100 MHz, $CDCl_3$) δ 164.0 (dd, *J* = 11.9 Hz, *J* = 254.4 Hz), 160.4 (dd, *J* = 12.3 Hz, *J* = 258.6 Hz), 134.5 (d, *J* = 5.5 Hz), 132.1 (dd, *J* = 2.8 Hz, *J* = 10.1 Hz), 117.6 (dd, *J* = 4.1 Hz, *J* = 11.3 Hz), 111.8 (dd, *J* = 3.8 Hz, *J* = 21.8 Hz), 105.1 (t, *J* = 25.8 Hz) ppm [11].

(*Z*)-*N*-hydroxypyridine-3-carbonimidoyl chloride (25o): orange solid, 38%, R_f = 0.38 (CyH/EtOAc 3:1). ^{1}H NMR (300 MHz, MeOD) δ 8.95 (s, 1H), 8.56 (d, *J* = 4.6 Hz, 1H), 8.25 – 8.21 (m, 1H), 7.48 (dd, *J* = 4.9 Hz, *J* = 8.1 Hz, 1H) ppm; ^{13}C NMR (100 MHz, MeOD) δ 152.8, 150.9, 147.8, 136.5, 130.1, 125.2 ppm [18].

(*Z*)-*N*-hydroxycyclopropanecarbonimidoyl chloride (25p): colorless oil, 66%, R_f = 0.52 (CyH/EtOAc 3:1). ^{1}H NMR (300 MHz, $CDCl_3$) δ 8.50 (s, 1H), 1.95 – 1.86 (m, 1H), 0.99 – 0.92 (m, 2H), 0.90 – 0.81 (m, 2H) ppm; ^{13}C NMR (100 MHz, $CDCl_3$) δ 144.4, 15.9, 5.9 ppm [19].

(*Z*)-*N*-hydroxyoxolane-3-carbonimidoyl chloride (25q): colorless oil, 52%, R_f = 0.52 (CyH/EtOAc 3:1). ^{1}H NMR (300 MHz, $CDCl_3$) δ 9.17 (s, 1H), 4.02 – 3.82 (m, 4H), 3.39 – 3.29 (m, 1H), 2.22 – 2.15 (m, 2H) ppm; ^{13}C NMR (100 MHz, $CDCl_3$) δ 141.5, 70.3, 68.2, 45.7, 30.1 ppm [20].

(*Z*)-*N*-hydroxycyclohexanecarbonimidoyl chloride (25r): colorless oil, 85%, R_f = 0.36 (CyH/EtOAc 3:1). ^{1}H NMR (300 MHz, $CDCl_3$) δ 8.56 (s, 1H), 2.46 (tt, *J* = 3.4 Hz, *J* = 11.4, 1H), 1.96 – 1.92 (m, 2H), 1.83 – 1.78 (m, 2H), 1.71 – 1.66 (m, 1H), 1.50 – 1.35 (m, 2H), 1.35 – 1.23 (m, 3H) ppm; ^{13}C NMR (100 MHz, $CDCl_3$) δ 146.5, 45.4, 30.2, 25.6, 25.5 ppm [21].

Characterization of 32b, 32s-z

***N*-benzyl-5-{1,4-dioxaspiro[4.5]decan-2-yl}-1,2-oxazole-3-carboxamide (32b):** yellowish solid, 68%, R_f = 0.39 (CyH/EtOAc 3:1), UHPLC-ESI-MS: R_t = 3.04, *m/z* = 343.2 $[M + H]^+$. ^{1}H NMR (300 MHz, $CDCl_3$) δ 7.36 – 7.29 (m, 5H), 7.09 (s br, 1H), 6.75 (s, 1H), 5.22 (t, *J* = 6.0 Hz, 1H), 4.62 (d, *J* = 6.0 Hz, 2H), 4.34 (dd, *J* = 6.6 Hz, *J* = 8.6 Hz, 1H), 4.09 (dd, *J* = 5.4 Hz, *J* = 8.6 Hz, 1H), 1.71 – 1.60 (m, 8H), 1.46 – 1.42 (m, 2H) ppm; ^{13}C NMR (100 MHz, $CDCl_3$) δ 173.3, 158.5, 140.4, 137.2, 128.8, 127.9, 127.8, 111.9, 101.9, 69.8, 68.1, 43.5, 35.8, 34.9, 25.0, 23.9, 23.8 ppm.

5-{1,4-dioxaspiro[4.5]decan-2-yl}-*N*-(4-fluorophenyl)- 1,2-oxazole-3-carboxamide (32s): yellowish solid, 58%, R_f = 0.53 (CyH/EtOAc 3:1), UHPLC-ESI-MS: R_t = 3.14, *m/z* = 347.2 $[M + H]^+$. ^{1}H NMR (300 MHz, $CDCl_3$) δ 8.48 (s, 1H), 7.64 – 7.59 (m, 2H), 7.10 – 7.04 (m, 2H), 6.80 (s, 1H), 5.26 (t, *J* = 6.0 Hz, 1H), 4.37 (dd, *J* = 6.6 Hz, *J* = 8.7 Hz, 1H), 4.12 (dd, *J* = 5.4 Hz, *J* = 8.7 Hz, 1H), 1.74 – 1.62 (m, 8H), 1.47 – 1.43 (m, 2H) ppm; ^{13}C NMR (100 MHz, $CDCl_3$) δ 173.9, 161.4, 157.5 (d, *J* = 171.1 Hz), 156.3, 132.9 (d, *J* = 2.8 Hz), 121.8 (d, *J* = 7.9 Hz), 115.9 (d, *J* = 22.6 Hz), 112.0, 101.9, 69.8, 68.1, 35.9, 34.9, 25.0, 23.9, 23.8 ppm.

5-{1,4-dioxaspiro[4.5]decan-2-yl}-*N*-[(thiophen-2-yl)methyl]-1,2-oxazole-3-carboxamide (32t): yellowish solid, 53%, R_f = 0.39 (CyH/EtOAc 3:1), UHPLC-ESI-MS: R_t = 3.00, *m/z* = 349.2 $[M + H]^+$. ^{1}H NMR (300 MHz, $CDCl_3$) δ 7.25 (dd, *J* = 1.2 Hz, *J* = 3.9 Hz, 1H), 7.12 (s br, 1H), 7.04 (dd, *J* = 1.0 Hz, *J* = 3.4 Hz, 1H), 6.97 (dd, *J* = 3.5 Hz, *J* = 5.1 Hz, 1H), 6.74 (s, 1H), 5.22 (t, *J* = 5.8 Hz, 1H), 4.79 (d, *J* = 5.9 Hz, 2H), 4.34 (dd, *J* = 6.6 Hz, *J* = 8.6 Hz, 1H), 4.09 (dd, *J* = 5.4 Hz, *J* = 8.6 Hz, 1H), 1.71 – 1.61 (m, 8H), 1.46 – 1.42 (m, 2H) ppm; ^{13}C NMR (100 MHz, $CDCl_3$) δ 173.4, 158.3, 143.3, 139.6, 127.0, 126.5, 125.6, 111.9, 101.8, 69.8, 68.1, 38.1, 35.8, 34.9, 25.0, 23.9, 23.8 ppm.

5-{1,4-dioxaspiro[4.5]decan-2-yl}-*N*-[(pyridin-3-yl)methyl]-1,2-oxazole-3-carboxamide (32u): yellow oil, 57%, R_f = 0.38 ($CHCl_3$/MeOH 5:1), UHPLC-ESI-MS: R_t = 2.00, *m/z* = 344.2 $[M + H]^+$. ^{1}H NMR (300 MHz, $CDCl_3$) δ 8.61 (d, *J* = 1.7 Hz, 1H), 8.56 (dd, *J* = 1.4 Hz, *J* = 4.8 Hz, 1H), 7.75 – 7.71 (m, 1H), 7.32 (dd, *J* = 4.9 Hz, *J* = 7.8 Hz, 1H), 7.21 (s br, 1H), 6.74 (s, 1H), 5.23 (t, *J* = 5.7 Hz, 1H), 4.65 (d, *J* = 6.2 Hz, 2H), 4.35 (dd, *J* = 6.6 Hz, *J* = 8.6 Hz, 1H), 4.09 (dd, *J* = 5.4 Hz, *J* = 8.7 Hz, 1H), 1.71 – 1.62 (m, 8H), 1.46 – 1.42 (m, 2H) ppm; ^{13}C NMR (100 MHz, $CDCl_3$) δ 173.6, 163.3, 158.8, 158.1, 148.8, 136.0, 133.3, 123.8, 111.9, 101.8, 69.8, 68.1, 40.9, 35.8, 34.9, 24.9, 23.9, 23.8 ppm.

5-{1,4-dioxaspiro[4.5]decan-2-yl}-*N*-(2-methoxyethyl)-1,2-oxazole-3-carboxamide (32v): yellow oil, 52%, R_f = 0.25 (CyH/EtOAc 3:1), UHPLC-ESI-MS: R_t = 2.57, *m/z* = 311.2 $[M + H]^+$. ^{1}H NMR (300 MHz, $CDCl_3$) δ 6.70 (s, 1H), 5.22 (t, *J* = 6.0 Hz, 1H), 4.34 (dd, *J* = 6.6 Hz, *J* = 8.6 Hz, 1H), 4.08 (dd, *J* = 5.5 Hz, *J* = 8.6 Hz, 1H), 3.64 – 3.59 (m, 2H), 3.52 (dd, *J* = 2.8 Hz, *J* = 7.5 Hz, 2H), 3.37 (s, 3H), 1.70 – 1.61 (m, 8H), 1.45 – 1.41 (m, 2H), ppm; ^{13}C NMR (100 MHz, $CDCl_3$) δ 173.1, 158.7, 158.4, 111.8, 101.8, 70.7, 69.8, 68.1, 58.8, 39.2, 35.8, 34.9, 24.9, 23.8 (d, *J* = 4.2 Hz) ppm.

5-{1,4-dioxaspiro[4.5]decan-2-yl}-3-(pyrrolidine-1-carbonyl)-1,2-oxazole (32z): brown oil, 18%, R_f = 0.25 (CyH/EtOAc 3:1), UHPLC-ESI-MS: R_t = 2.79, *m/z* = 307.2 $[M + H]^+$. ^{1}H NMR (300 MHz, $CDCl_3$) δ 6.67 (s, 1H), 5.22 (t, *J* = 6.1 Hz, 1H), 4.34 (dd, *J* = 6.6 Hz, *J* = 8.6 Hz, 1H), 4.09 (dd, *J* = 5.7 Hz, *J* = 8.6 Hz, 1H), 3.85 (t, *J* = 6.5 Hz, 2H), 3.65 (t, *J* = 6.6 Hz, 2H), 1.99 – 1.91 (m, 4H), 1.74– 1.62 (m, 8H), 1.45 – 1.41 (m, 2H) ppm; ^{13}C NMR (100 MHz, $CDCl_3$) δ 171.4, 159.7, 158.4, 111.8, 103.4, 69.8, 68.2, 48.7, 46.9, 35.8, 35.0, 26.2, 25.0, 23.9, 23.8 ppm.

Biological activity of LsrK

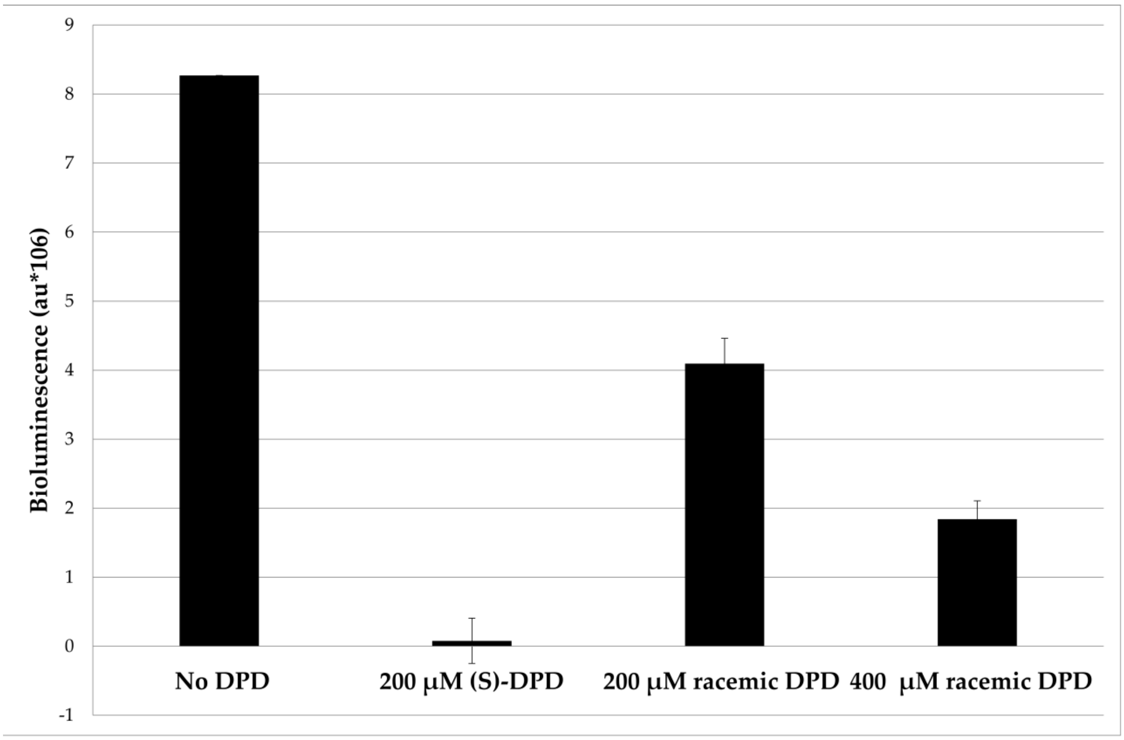

Figure S1: Activity of LsrK in the presence of racemic DPD and (*S*)-DPD (from OMM Scientific) detected by measuring ATP depletion.

Table (biology)

Series	Structure	Compound	R[1]	Inhibition (%)
**Series I** *1,4-disubstituted 1,2,3-triazoles DPD-derivatives*	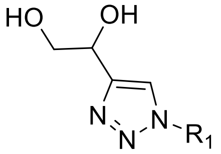	**18a**	$(CH_2)_2$-Ph	0
		**18b**	(CH_2)-Ph	1.9
		**18c**	$(CH_2)_2$-*o*-F-Ph	3.3
		**18d**	$(CH_2)_2$-*m*-Pyr	0.1
		**18e**	$(CH_2)_5$-CN	2.2
		**18f**	$(CH_2)_2$-CyH	2.7
		**18g**	H	2.3
		**18h**	CH_3	2.0
		**18i**	CH_2-Cyp	2.5
		**18j**	*n*-Bu	5.5
		**18k**	$(CH_2)_2$-O-CH_3CH_2	2.2
**Series II** *1,5-disubstituted 1,2,3-triazoles DPD-derivatives*	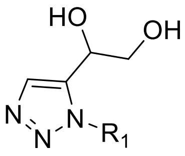	**19a**	$(CH_2)_2$-Ph	2.0
		**19h**	CH_3	2.5
		**19i**	CH_2-Cyp	1.6
		**19j**	*n*-Bu	2.4
		**19k**	$(CH_2)_2$-O-CH_3CH_2	2.0
**Series III** *3,5-disubstituted isoxazoles DPD-derivatives*	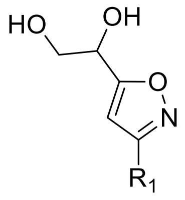	**26l**	*p*-CH_3-Ph	1.9
		**26m**	*m*-Cl-Ph	0.1
		**26n**	*o, p*-di-F-Ph	0.2
		**26o**	*m*-Pyr	4.8
		**26p**	Cyp	8.4
		**26q**	*m*-THF	0.8
		**26r**	CyH	5.5
**Series IV** *3,5-disubstituted isoxazoles DPD-derivatives*	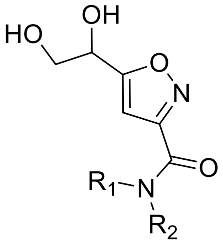	**33b**	CH_2-Ph	2.5
		**33s**	*p*-F-Ph	2.5
		**33t**	CH_2-thiophene	2.5
		**33u**	CH_2-*m*-Pyr	1.2
		**33v**	$(CH_2)_2$-O-CH_3	1.0
		**33z**	Pyrrolidine	2.5
**Other**	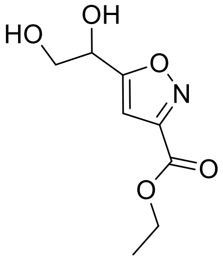	**29**	—	3.1
**Other**	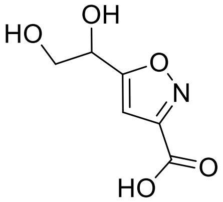	**31**	—	2.1

**Other**	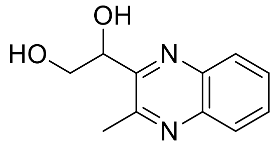	**Quinoxaline-DPD**	—	10.9
**Other**	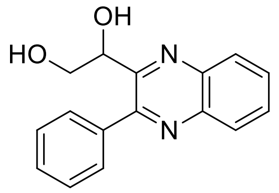	**Quinoxaline-Ph-DPD**	—	0

Table S3: Biological activity of the synthesized compounds. All compounds have been tested at 200 μM

References

1. Colombano, G.; Travelli, C.; Galli, U.; Caldarelli, A.; Chini, M. G.; Canonico, P. L.; Sorba, G.; Bifulco, G.; Tron, G. C.; Genazzani, A. A. A Novel Potent Nicotinamide Phosphoribosyltransferase Inhibitor Synthesized via Click Chemistry. *J. Med. Chem.* **2010**, *53*, 616–623, doi:10.1021/jm9010669.
2. Campbell-Verduyn, L. S.; Mirfeizi, L.; Dierckx, R. A.; Elsinga, P. H.; Feringa, B. L. Phosphoramidite accelerated copper(I)-catalyzed [3 + 2] cycloadditions of azides and alkynes. *Chem. Commun.* **2009**, 2139–2141, doi:10.1039/B822994E.
3. Suzuki, T.; Ota, Y.; Ri, M.; Bando, M.; Gotoh, A.; Itoh, Y.; Tsumoto, H.; Tatum, P. R.; Mizukami, T.; Nakagawa, H.; Iida, S.; Ueda, R.; Shirahige, K.; Miyata, N. Rapid Discovery of Highly Potent and Selective Inhibitors of Histone Deacetylase 8 Using Click Chemistry to Generate Candidate Libraries. *J. Med. Chem.* **2012**, *55*, 9562–9575, doi:10.1021/jm300837y.
4. Bevilacqua, V.; King, M.; Chaumontet, M.; Nothisen, M.; Gabillet, S.; Buisson, D.; Puente, C.; Wagner, A.; Taran, F. Copper-Chelating Azides for Efficient Click Conjugation Reactions in Complex Media. *Angew. Chem. Int. Ed.* **2014**, *53*, 5872–5876, doi:10.1002/anie.201310671.
5. Luo, L.; Wilhelm, C.; Sun, A.; Grey, C. P.; Lauher, J. W.; Goroff, N. S. Poly(diiododiacetylene): Preparation, Isolation, and Full Characterization of a Very Simple Poly(diacetylene). *J. Am. Chem. Soc.* **2008**, *130*, 7702–7709, doi:10.1021/ja8011403.
6. Wijtmans, M.; de Graaf, C.; de Kloe, G.; Istyastono, E. P.; Smit, J.; Lim, H.; Boonnak, R.; Nijmeijer, S.; Smits, R. A.; Jongejan, A.; Zuiderveld, O.; de Esch, I. J. P.; Leurs, R. Triazole Ligands Reveal Distinct Molecular Features That Induce Histamine H4 Receptor Affinity and Subtly Govern H4/H3 Subtype Selectivity. *J. Med. Chem.* **2011**, *54*, 1693–1703, doi:10.1021/jm1013488.
7. MacDonald, J. P.; Badillo, J. J.; Arevalo, G. E.; Silva-García, A.; Franz, A. K. Catalytic Stereoselective Synthesis of Diverse Oxindoles and Spirooxindoles from Isatins. *ACS Comb. Sci.* **2012**, *14*, 285–293, doi:10.1021/co300003c.
8. Shao, C.; Wang, X.; Zhang, Q.; Luo, S.; Zhao, J.; Hu, Y. Acid–Base Jointly Promoted Copper(I)-Catalyzed Azide–Alkyne Cycloaddition. *J. Org. Chem.* **2011**, *76*, 6832–6836, doi:10.1021/jo200869a.
9. Himo, F.; Lovell, T.; Hilgraf, R.; Rostovtsev, V. V.; Noodleman, L.; Sharpless, K. B.; Fokin, V. V. Copper(I)-Catalyzed Synthesis of Azoles. DFT Study Predicts Unprecedented Reactivity and Intermediates. *J. Am. Chem. Soc.* **2005**, *127*, 210–216, doi:10.1021/ja0471525.
10. Allen, C. L.; Davulcu, S.; Williams, J. M. J. Catalytic Acylation of Amines with Aldehydes or Aldoximes. *Org. Lett.* **2010**, *12*, 5096–5099, doi:10.1021/ol101978h.
11. Bonjouklian, R.; Johnson, D. W.; Lander, P. A.; Lohman, M. C.; Patel, V. F.; Vepachedu, S.; Xie, Y. Compounds and method for inhibiting MRP1 2003.
12. Erenler, R. Synthesis and Characterization of Pyridyl Propargyloximes. *Asian J. Chem.* **2011**, *23*, 3546–3548.
13. Wu, P.-L.; Wang, W.-S. Thermal Ring-Expansion of N-Acyl Cyclopropyl Imines. *J. Org. Chem.* **1994**, *59*, 622–627, doi:10.1021/jo00082a020.
14. Metzner, R.; Okazaki, S.; Asano, Y.; Gröger, H. Cyanide-free Enantioselective Synthesis of Nitriles: Synthetic Proof of a Biocatalytic Concept and Mechanistic Insights. *ChemCatChem* **2014**, *6*, 3105–3109, doi:10.1002/cctc.201402612.
15. Minakata, S.; Okumura, S.; Nagamachi, T.; Takeda, Y. Generation of Nitrile Oxides from Oximes Using *t* -BuOI and Their Cycloaddition. *Org. Lett.* **2011**, *13*, 2966–2969, doi:10.1021/ol2010616.
16. Tóth, M.; Kun, S.; Bokor, É.; Benltifa, M.; Tallec, G.; Vidal, S.; Docsa, T.; Gergely, P.; Somsák, L.; Praly, J.-P. Synthesis and structure–activity relationships of C-glycosylated oxadiazoles as inhibitors of glycogen phosphorylase. *Bioorg. Med. Chem.* **2009**, *17*, 4773–4785, doi:10.1016/j.bmc.2009.04.036.
17. Hanan, E. J.; van Abbema, A.; Barrett, K.; Blair, W. S.; Blaney, J.; Chang, C.; Eigenbrot, C.; Flynn, S.; Gibbons, P.; Hurley, C. A.; Kenny, J. R.; Kulagowski, J.; Lee, L.; Magnuson, S. R.; Morris, C.; Murray, J.; Pastor, R. M.; Rawson, T.; Siu, M.; Ultsch, M.; Zhou, A.; Sampath, D.; Lyssikatos, J. P. Discovery of Potent and Selective Pyrazolopyrimidine Janus Kinase 2 Inhibitors. *J. Med. Chem.* **2012**, *55*, 10090–10107, doi:10.1021/jm3012239.

18. Zhu, J.; Ye, Y.; Ning, M.; Mándi, A.; Feng, Y.; Zou, Q.; Kurtán, T.; Leng, Y.; Shen, J. Design, Synthesis, and Structure–Activity Relationships of 3,4,5-Trisubstituted 4,5-Dihydro-1,2,4-oxadiazoles as TGR5 Agonists. *ChemMedChem* **2013**, *8*, 1210–1223, doi:10.1002/cmdc.201300144.
19. Samajdar, S.; ABBINENI, C.; SASMAL, S.; Hosahalli, S. Bicyclic heterocyclic derivatives as bromodomain inhibitors 2015.
20. US20090156603A1 - 2-aminopyridine analogs as glucokinase activators - Google Patents Available online: https://patents.google.com/patent/US20090156603/ko
21. WO2006090234A1 - Heterocyclic derivatives as cell adhesion inhibitors - Google Patents Available online: https://patents.google.com/patent/WO2006090234A1/zh:
